# Combination of an autophagy inhibitor with immunoadjuvants and an anti-PD-L1 antibody in multifunctional nanoparticles for enhanced breast cancer immunotherapy

**DOI:** 10.1186/s12916-022-02614-8

**Published:** 2022-10-28

**Authors:** Yibin Cheng, Caixia Wang, Huihui Wang, Zhiwei Zhang, Xiaopeng Yang, Yanming Dong, Lixin Ma, Jingwen Luo

**Affiliations:** 1 State Key Laboratory of Biocatalysis and Enzyme Engineering, Hubei Key Laboratory of Industrial Biotechnology, Hubei Collaborative Innovation Center for Green Transformation of Bio-Resources, School of Life Sciences, Hubei University, Wuhan, 430062 P. R. China; 2 Key Laboratory for the Synthesis and Application of Organic Functional Molecules, College of Chemistry and Chemical Engineering, Hubei University, Wuhan, 430062 P. R. China

**Keywords:** Immuno-chemotherapy, Anti-PD-L1 antibody, Multifunctional nanoparticles, Autophagy response

## Abstract

**Background:**

The application of combination therapy for cancer treatment is limited due to poor tumor-specific drug delivery and the abscopal effect.

**Methods:**

Here, PD-L1- and CD44-responsive multifunctional nanoparticles were developed using a polymer complex of polyethyleneimine and oleic acid (PEI-OA) and loaded with two chemotherapeutic drugs (paclitaxel and chloroquine), an antigen (ovalbumin), an immunopotentiator (CpG), and an immune checkpoint inhibitor (anti-PD-L1 antibody).

**Results:**

PEI-OA greatly improved the drug loading capacity and encapsulation efficiency of the nanoplatform, while the anti-PD-L1 antibody significantly increased its cellular uptake compared to other treatment formulations. Pharmacodynamic experiments confirmed that the anti-PD-L1 antibody can strongly inhibit primary breast cancer and increase levels of CD4+ and CD8+ T cell at the tumor site. In addition, chloroquine reversed the “immune-cold” environment and improved the anti-tumor effect of both chemotherapeutics and immune checkpoint inhibitors, while it induced strong immune memory and prevented lung metastasis.

**Conclusions:**

Our strategy serves as a promising approach to the rational design of nanodelivery systems for simultaneous active targeting, autophagy inhibition, and chemotherapy that can be combined with immune-checkpoint inhibitors for enhanced breast cancer treatment.

**Supplementary Information:**

The online version contains supplementary material available at 10.1186/s12916-022-02614-8.

## Background

Chemo-immunotherapy is considered a major strategy for the treatment of established solid tumors. In recent years, several methods have been developed for treating post-surgery residual tumors and preventing tumor recurrence and metastasis, including a combination of chemotherapeutics and cancer vaccines, checkpoint therapies, and adoptive T-cell transfer [1–4]. Although combination therapy is more effective than individual therapies, it cannot meet current clinical needs due to the presence of few T cells (“immune-cold” tumors) and a high number of immunosuppressive cells in the tumor microenvironment (TME), including myeloid-derived suppressor cells, tumor-associated macrophages, and regulatory T cells [5].

Recent studies have shown that autophagy in the TME plays a dual role [6, 7]: it can inhibit as well as promote tumor growth by regulating the immune response [8] and the survival, apoptosis, differentiation, activation, effector function, and metastasis of immune cells [9]. For example, autophagy promotes the survival and differentiation of T cells in the TME [10], while autophagy of naive T cells protects them from mitochondrial apoptosis induced by reactive oxygen [11]. High levels of lactic acid in tumors may interfere with naive T cell autophagy and weaken anti-tumor response [12]. Moreover, increased apoptosis and functional defects of autophagy-deficient regulatory T cells can enhance tumor resistance [13]. Therefore, targeting autophagy may reshape the TME and reverse its “immune-cold” character. For instance, autophagy inhibition can upregulate Th1 chemokines and promote the infiltration of effector immune cells at the tumor site [14]. In addition, it can enhance anti-tumor T cell responses by improving antigen presentation and processing [15], while it can upregulate major histocompatibility complex (MHC) class I molecules on the cell surface and prevent the autophagic degradation of granzyme B, thereby allowing cell lysis [16, 17].

Chloroquine (CQ) is a typical autophagy inhibitor that has been used along with various treatments in multiple clinical trials for anticancer treatment [18–20]. A recent study on a mouse model of pancreatic cancer has shown that treatment with CQ combined with dual immune-checkpoint blockade therapy using anti-PD-L1 and anti-CTLA4 antibodies can effectively inhibit autophagy-mediated MHC-I degradation and enhance antitumor immune responses [21]. These results suggest that autophagy targeting combined with chemo-immunotherapy using integrative multifunctional nanoparticles (MNPs), which are generally known for their excellent stability, biocompatibility, and encapsulation efficiency [22], may improve the therapeutic potential of individual treatments. Nevertheless, the successful design of MNPs requires an appropriate way to add immunoadjuvants, since the separate administration of chemotherapeutics, cancer antibodies, antigens, and immunopotentiators may lead to severe side effects and non-specific, systemic immune responses [23]. Given also that most cancer vaccines consisting of cancer antigens and immunopotentiators have low efficiency due to initial burst and off-target release, it is clear that adjuvants should show (1) high encapsulation efficiency for cancer chemotherapeutics, antigens, and immunopotentiators in order to prevent initial burst release and promote controlled release; (2) enhanced targeted delivery to antigen-presenting and tumor cells; (3) ability to induce effective anti-tumor T-cell responses; and (4) good biocompatibility.

**Fig. 1:**
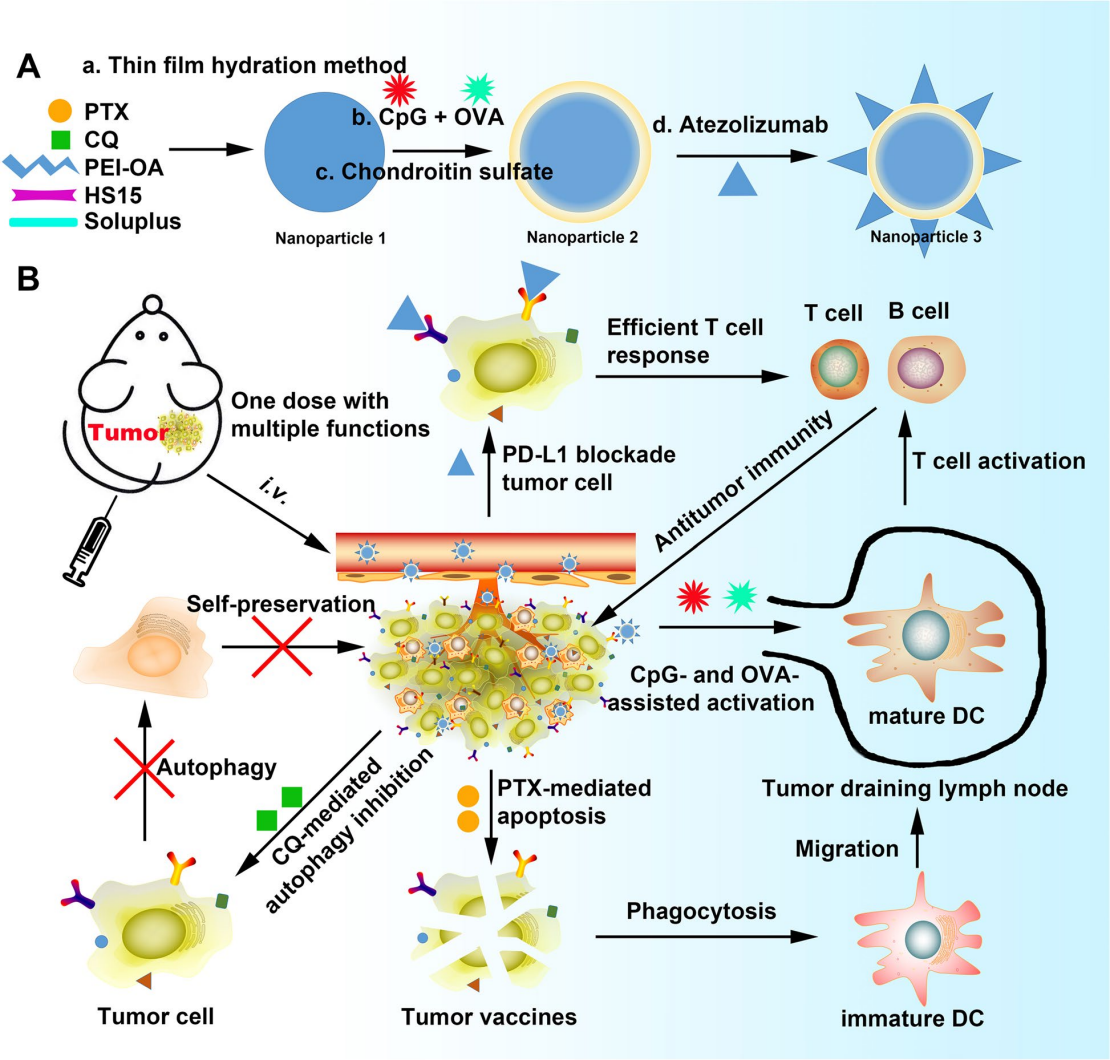
Schematic illustration of the combined application of chemotherapy, immunotherapy, and PD-L1 blockade therapy in tumor-bearing mice. CpG, immunopotentiator; CQ, chloroquine, DC, dendritic cell; HS15, Solutol HS15®; OVA, ovalbumin; PEI-OA, polyethyleneimine-oleic acid polymer; PTX, paclitaxel

In this study, we describe an ultrafast, convenient, and universal self-assembly route for the preparation of an integrative multilayered nanoplatform using immunoadjuvants for targeted tumor and lymph node delivery and durable antitumor immunity. The developed MNPs were loaded with two chemotherapeutic drugs [paclitaxel (PTX) and CQ], an antigen [ovalbumin (OVA)], an immunopotentiator (CpG), and an immune checkpoint inhibitor (anti-PD-L1 antibody, also known as atezolizumab). We found that atezolizumab neutralized PD-L1 and activate immune responses, while acting synergistically with chondroitin sulfate (CS) on the outmost layer of the nanoparticles to target PD-L1 and CD44 for combined autophagy modulation and cancer immunotherapy. The described nanoplatform also efficiently delivered PTX and CQ to tumor cells as well as OVA and CpG to draining lymph nodes in a targeted and controlled manner, thus enhancing their availability and limiting off-target effects. Furthermore, the combination of CQ with atezolizumab reversed the immunosuppressive TME, elicited strong tumor antigen-specific immune responses, and induced sustained tumor suppression in tumor-bearing mice (Fig. 1).

## Methods

### Materials

OVA was purchased from Sigma-Aldrich (St Louis, MO, USA). Polyethyleneimine (PEI, molecular weight 1800 Da) and oleic acid (OA) were obtained from Shanghai Aladdin Biochemical Technology Co., Ltd (Shanghai, China). PTX and CQ were purchased from Dalian Meilun Biotech Co., Ltd. (Dalian, China). 4-Chlorobenzenesulfonate salt (DiD) was provided by Biotium (Hayward, CA, USA). Fluorescein (FITC)-conjugated rat anti-mouse CD4 antibody, PE-conjugated rat anti-mouse CD8a antibody, and the Annexin V-FITC/PI apoptosis detection kit were purchased from BioLegend, Inc. (San Diego, CA, USA). Anti-GAPDH was obtained from CWBIO (Beijing, China), and monoclonal anti-LC3B antibodies were purchased from Sigma-Aldrich. Anti-p62 (SQSTM1) antibodies were from Proteintech Group (Rosemont, IL, USA), and horseradish peroxidase (HRP)-conjugated-goat anti-rabbit IgG (H + L) secondary antibody was from Invitrogen (catalog no. 31460, Carlsbad, CA, USA). FITC-conjugated ImmunoPure goat anti-rabbit IgG (H + L) antibodies were purchased from Feiyi Technology (Wuhan, China), while anti-PD-L1 antibody (atezolizumab) was from Selleck Chemicals (Houston, TX, USA). Mouse mammary breast tumor cells (4T1) and Hela cells were obtained from the Chinese Academy of Science Cell Bank (Shanghai, China).

### Animals

Healthy female BALB/c mice (6–8 weeks) were obtained from Wuhan Institute of Virology (Wuhan, China). All animal experiments were approved by the Ethics Committee of Wuhan Institute of Virology (Approval number: WIVA04202102).

### Synthesis and characterization of polymers

PEI (0.05 mmol, 1800 Da) was dissolved in 10 mL of methanol, and 0.8 mmol of 1-ethyl-3-(3-dimethylaminopropyl) carbodiimide hydrochloride was added. After stirring for 10 min, a solution of 0.8 mmol OA in 10 mL methanol was slowly added using a constant-pressure dropping funnel, and the reaction mixture was stirred at room temperature overnight under nitrogen. The mixture was then transferred to a dialysis bag with a molecular-weight cutoff (MWCO) of 3500 Da and dialyzed against a 1:1 water/methanol mixture for 24 h, then against purified water for another two days. The obtained oleic acid-modified polyethyleneimine (PEI-OA) material was ultimately freeze-dried, weighed, and stored at −20 °C. ^1^H NMR spectra were recorded in dimethyl sulfoxide on a Varian UNITY INOVA400 NMR spectrometer (Palo Alto, CA, USA).

### Preparation and characterization of MNPs

Nanoparticles 1 were prepared by the thin-film hydration method. PEI-OA, Soluplus and Solutol HS15® (HS15) were first dissolved in methanol at a molar ratio of 1:6:2. Soluplus and HS15 were the basic materials for nanoparticles preparation. They were combined to make nanoparticles possess uniform and suitable particle size. PEI-OA was added to give nanoparticle-positive charge. After removing the organic solvent by rotary evaporation at 37 °C, the obtained thin film was hydrated with 5% glucose solution at 60 °C to form nanoparticles 1. Next, nanoparticles 1 were added to a solution of OVA (150 μg/mL), CpG (50 μg/mL), and CS (16 mg/mL) in 5% glucose and stirred gently to give nanoparticles 2, which were then rapidly mixed with a solution of atezolizumab (2 mg/mL) using a vortex shaker for 1 min at room temperature to afford the desired MNPs (3). Unencapsulated drug was removed by ultrafiltration over a membrane (MWCO 1000 kDa, Spectrum Laboratories, CA, USA), and nanoparticles were diluted to 1:100 in 5% glucose solution, transferred to disposable polystyrene cuvettes, and analyzed using a Zetasizer Nano ZSP (Malvern Instruments, Worcestershire, UK). In general, the ratio of PTX: CQ: PEI-OA: Soluplus: HS15: OVA: CpG: CS:atezolizumab was 200:40:300:1800:600:3:1:1600:100.

To confirm that the nanoparticles could be loaded simultaneously with chemotherapeutic drugs and biomolecules, MNPs were incubated with DiD- and/or FITC-labeled OVA, then analyzed with a high-sensitivity flow cytometer (Beckman Coulter, Brea, CA, USA) equipped with three single-photon-counting avalanche photodiode detectors for the simultaneous detection of side scattering and two-color fluorescence. Flow cytometric data were analyzed using FlowJo™ v10.0 (Tree Star, Inc., Ashland, OR, USA).

### Drug loading capacity and encapsulation efficiency

Isolated, unencapsulated drugs were redissolved in methanol and detected by HPLC (Agilent, Santa Clara, CA, USA). Loading capacity and encapsulation efficiency were calculated as follows:

1 $$\textrm{Loading}\ \textrm{capacity}\ \left(\%\right)=\frac{\textrm{amount}\ \textrm{of}\ \textrm{drug}\ \textrm{in}\ \textrm{nanoparticles}}{\textrm{total}\ \textrm{mass}\ \textrm{of}\ \textrm{nanoparticles}}\times 100$$

2 $$\textrm{Encapsulation}\ \textrm{efficiency}\ \left(\%\right)=\frac{\textrm{amount}\ \textrm{of}\ \textrm{drug}\ \textrm{in}\ \textrm{nanoparticles}}{\textrm{total}\ \textrm{drug}\ \textrm{dose}}\times 100$$

OVA- and CpG-loaded MNPs were also purified by ultrafiltration, and their content was determined using the bicinchoninic acid (BCA) protein assay kit (Pierce, Rockford, IL, USA) and electrophoresis on agarose gel (1%) stained with GoldView (Generay Biotech, Shanghai, China).

### Chromatographic conditions

Chromatographic analysis was performed on an Agilent 1290 liquid chromatography system with an Agilent C18 column (250 mm × 4.6 mm, 5 μm). PTX concentrations were measured at 227 nm using methanol/water (65:35) as the mobile phase. CQ concentrations were measured at 343 nm using a mixture of acetonitrile, methanol, water, and ammonium hydroxide (78:20:35:1.65) as the mobile phase. The flow rate was 1.0 mL/min and the column temperature was 40 °C.

### Cytotoxicity

4T1 cells were seeded in 96-well plates (5 × 10^3^ cells per well in 100 μL). After 12 h, cells were treated with fresh medium (control) or culture medium containing different concentrations of blank nanoparticles (N), PTX-S, CpG+OVA-S, CpG+OVA+PTX-S, PTX-N, CpG+OVA-N, CpG+OVA+PTX-N, CpG+OVA+PTX+CQ-N, or CpG+OVA+PTX+CQ-N/A (PTX, 0.25–40 μg/mL; CQ, 0.05–8 μg/mL). Cell proliferation after incubation with different formulations for 24 h was determined using the 3-(4,5-dimethyl-2-thiazolyl)-2,5-diphenyl-2H-tetrazolium bromide (MTT) assay (Sigma-Aldrich, St Louis, MO, USA).

### Apoptosis

4T1 cells were seeded in 12-well plates (10^5^ cells/well). After 48 h, cells were incubated with blank nanoparticles (N), PTX-S, CpG+OVA-S, CpG+OVA+PTX-S, PTX-N, CpG+OVA-N, CpG+OVA+PTX-N, CpG+OVA+PTX+CQ-N, or CpG+OVA+PTX+CQ-N/A for 48 h. The final concentration of PTX was 1 μg/mL and that of CQ was 0.2 μg/mL. At 48 h post-incubation, the treated cells were stained using the Annexin V-FITC/PI apoptosis detection kit and analyzed by flow cytometry (Becton, Dickinson and Company, Franklin Lakes, NJ, USA).

### Cellular uptake

4T1 cells were seeded in six-well plates (3 × 10^5^ cells/well). After 24 h, DiD+FAM-CpG-S or DiD+FAM-CpG-N/A (both with a DiD concentration of 1 μg/mL) was added and cells were incubated for 2 or 4 h, respectively. Afterward, the cells were digested, collected, and analyzed by flow cytometry (Beckman Coulter).

4T1 cells were seeded in six-well plates with a coverslip in each well and incubated with DiD+FAM-CpG-S or DiD+FAM-CpG-N/A as above. At 2 or 4 h post-treatment, cells were fixed with 4% polyformaldehyde, washed twice with phosphate-buffered saline (PBS), and stained with DAPI (0.5 μg/mL; Biofroxx, Einhausen, Germany) for 5 min. The fluorescence intensity was observed with a Nikon A1R+ confocal microscope (Tokyo Metropolis, Japan).

### Cell culture and transfection

4T1 and HeLa cells were cultured in RPMI-1640/ Dulbecco’s modified Eagle medium (DMEM) (HyClone, Logan, UT, USA) with 10% fetal bovine serum (PAN-Biotech, Aidenbach, Germany). HeLa cells were transfected with mCherry-GFP-LC3B using Lipofectamine 2000 (Invitrogen) and cultured with different formulations in RPMI-1640/DMEM for 4 h.

### Western blot analysis

Western blot analysis was performed as described [24]. Proteins were first extracted from HeLa cells or tumor samples. Whole extracts were then analyzed by sodium dodecyl sulfate-polyacrylamide gel electrophoresis and transferred to a 0.45-μm polyvinylidene fluoride membrane (Roche Diagnostics, IN, USA). Membranes were blocked with 5% bovine serum albumin (BSA) in TBST (20 mM Tris-HCl, pH 7.5, 150 mM NaCl, 0.1% Tween-20), incubated with primary antibodies at 4 °C overnight and then with the HPR-labeled secondary antibody. Proteins were detected using an enhanced chemiluminescent reagent kit (Millipore, Billerica, MA, USA) and densitometry images were obtained using ImageJ software (vJ2, NIH, Maryland, US). Data were processed using Microsoft Excel.

### Immunofluorescence analysis

Immunofluorescence analysis was performed according to our previous study [25]. First, the tumor and/or major organ tissues were embedded in paraffin and cut into 2-μm sections using a slicer (Leica, Bensheim, Germany). 4T1 and HeLa cells cultured on glass coverslips were also treated with different preparations for 4 h. Then, the cells and tissue sections were fixed with 4% paraformaldehyde at 4 °C for 30 min and antigens were retrieved with an AR buffer (Leica, AR9961) and permeabilized with 0.1% Triton X-100 in PBS for 20 min. After blocking with 5% BSA at room temperature for at least 30 min, the samples were incubated with primary antibodies at 4 °C overnight. After washing three times with PBS, the samples were incubated with an indirect immunofluorescence secondary antibody at room temperature for 1 h, while cell nuclei were stained with DAPI for 5 min in the dark. Images were acquired with a confocal fluorescence microscope (ZEISS, LSM 980 with Airyscan 2, Oberkochen, Germany). For autophagy flux analysis, HeLa cells were transfected with mCherry-GFP-LC3B tandem reporter and imaged at 48 h post-transfection by confocal fluorescence microscopy.

### Tumor targeting ability

Female BALB/c mice were injected subcutaneously with 4T1 cells (100 μL; 2 × 10^5^ cells/mouse). On day 7, mice with a tumor volume of ~100 mm^3^ were randomly divided into two groups to receive DiD-S or DiD-N/A *via* intravenous injection (DiD, 1 mg/kg). The distribution of nanoparticles at 2, 4, 8, 24, and 48 h post-injection and the fluorescence intensity of major organs ex vivo were examined using a Lumina III Imaging System (PerkinElmer, MA, USA). Frozen tumor sections incubated with rabbit anti-mouse CD44 and PD-L1 antibodies and subsequently with FITC-labeled donkey anti-rabbit secondary antibody were also stained with DAPI and observed by confocal microscopy.

### In vivo antitumor effects

A model of 4T1 tumor-bearing BALB/c mice was established as described above. On day 5, mice were randomly divided into nine groups (*n* = 5) and injected intravenously twice with CpG+OVA+PTX-S, PTX-N, CpG+OVA-N, CpG+OVA+PTX-N, CpG+OVA+PTX-N/A, CpG+OVA+PTX+CQ-N, or CpG+OVA+PTX+CQ-N/A every 5 days. A 5% glucose solution was used as control. The PTX dose was 10 mg/kg, the CQ dose was 2 mg/kg, and the atezolizumab dose was 50 μg/mouse. The tumor size and body weight were measured every 2 days. At 5 days after the final injection, all mice were sacrificed and the tumors of each group were weighed, photographed, and sampled along with major organs for H&E staining.

### Immune cell activation and cytokine secretion

Freshly isolated tumor tissues were cut into pieces, digested with collagenase IV for 1 h, and filtered with cell strainers to prepare a single-cell suspension. About 2 × 10^7^ cells/sample were then incubated with different antibodies and the number of the corresponding T cells was determined by flow cytometry. T cells were detected using PE rat anti-mouse CD8α and FITC rat anti-mouse CD4 antibodies. Part of the tumor and spleen tissues were also fixed in paraffin for immunohistochemical staining. The levels of IFN-γ and TNF-α in the animal serum were analyzed with an ELISA kit (Sigma-Aldrich).

### Statistical analysis

Differences between multiple groups were analyzed for significance using one-way analysis of variance (ANOVA) followed by Tukey’s multiple comparisons test, while the two-tailed Student’s *t*-test was used for two groups. The results were presented as mean ± standard deviation (SD). Differences associated with ^*^P < 0.05 were considered statically significant.

## Results

### Design and characterization of MNPs

**Fig. 2:**
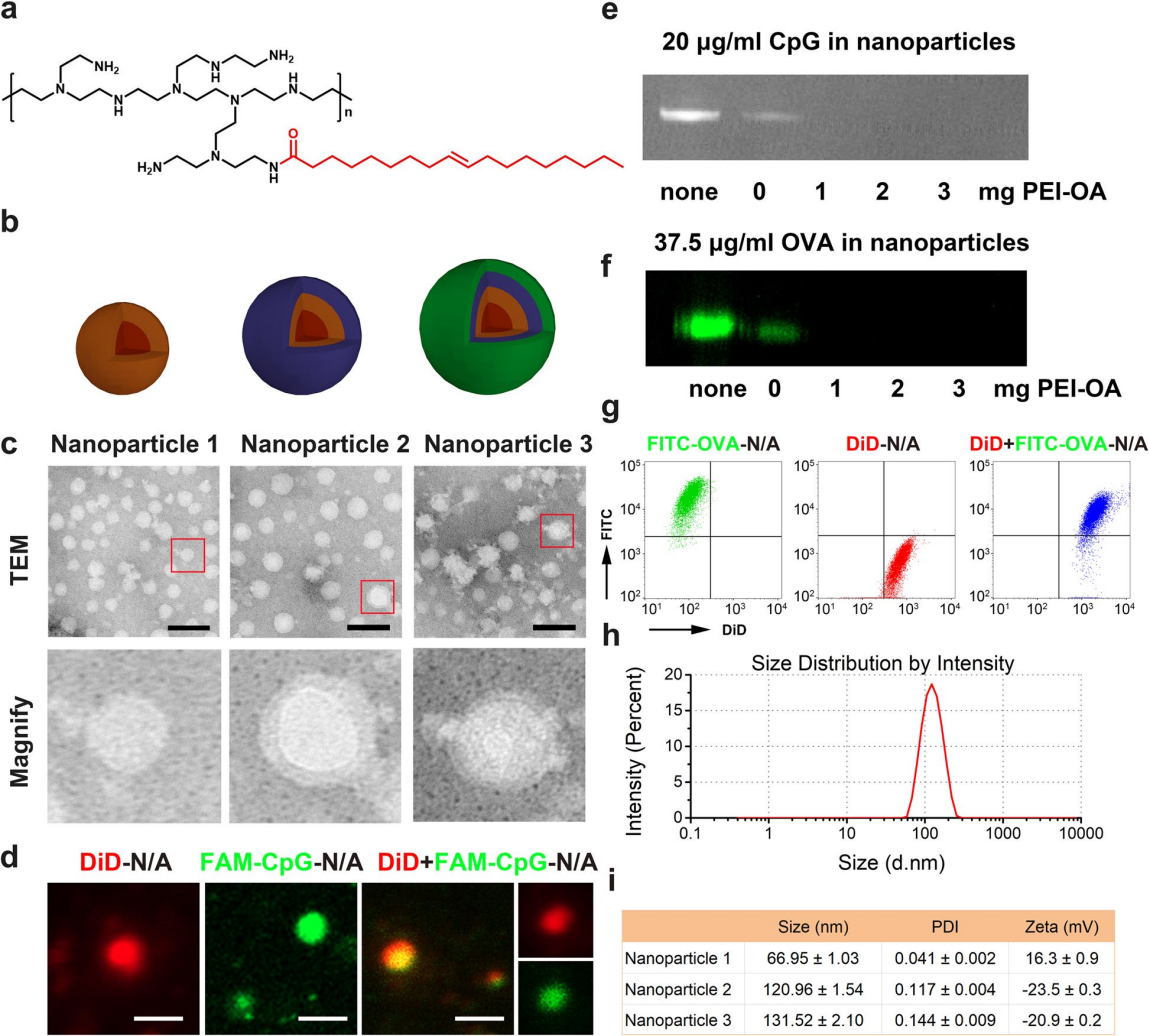
Construction and characterization of multifunctional nanoparticles (MNPs). **a** Chemical structure of the polyethyleneimine-oleic acid (PEI-OA) polymer. **b** Nanoparticles 1 were prepared by the thin-film hydration method. Encapsulation of an immunopotentiator (CpG) and ovalbumin (OVA) into 1 by electrostatic interactions, followed by surface coating with atezolizumab and chondroitin sulfate generated nanoparticles 3. **c** Transmission electron micrographs (TEM) of nanoparticles 1–3. Scale bar, 200 nm. **d** Confocal microscopic images of DiD-N/A, FAM-CpG-N/A, and DiD+FAM-CpG-N/A. Scale bar, 200 nm. **e** Agarose gel electrophoresis of DiD+FAM-CpG-N/A. **f** Western blot analysis of DiD-N/A, FITC-OVA-N/A, and DiD+FITC-OVA-N/A. Free FITC-OVA solution was used as control. **g** Flow cytometry of DiD-N/A, FITC-OVA-N/A, and DiD+FITC-OVA-N/A. **h** Representative size distribution of nanoparticles 3, as determined by dynamic light scattering. **i** Size, polydispersity index (PDI), and zeta potential of nanoparticles 1–3. Data are shown as mean ± SD (*n* = 4). DiD, 4-chlorobenzenesulfonate salt; FAM, fluorescein amidite; FITC, fluorescein; N, nanoparticles; S, solution

In order to construct a multifunctional treatment platform able to co-encapsulate small chemical drugs, nucleic acid, peptide and/or protein, we synthesized a polyethyleneimine-oleic acid polymer complex (PEI-OA) by conjugating OA to the carboxyl groups of PEI (molecular weight 1800 Da) (Fig. 2a). Nanoparticles 1 were then prepared by the thin-film hydration method using PEI-OA, HS15, and Soluplus (Fig. 1a). Next, nanoparticles 1 were added to a solution of CpG and OVA in 5% glucose to prepare nanoparticles 2. The positive charge of nanoparticles 1 was partially neutralized by the negative charge of CpG and OVA (Fig. 2b). The nanoparticle solution was then added to a CS solution to obtain completely negatively charged nanoparticles 2, which gave nanoparticles 3 after reaction with atezolizumab.

Scanning electron microscopy indicated that nanoparticles 2 and 3 were homogeneously spherical, but nanoparticles 3 had a rougher surface and a remarkably different size distribution and morphology (Fig. 2c), confirming that a layer of antibodies had adsorbed to the MNP surface. To verify that MNPs were generated through electrostatic interactions between nanoparticles 1 and CpG, nanoparticles 1 were incubated with fluorescein amidite-labeled CpG (FAM-CpG) and analyzed by confocal microscopy imaging. DiD was encapsulated in MNPs (DiD-N/A) and showed red fluorescence. FAM-CpG was loaded in MNPs and showed green fluorescence. The particles co-loaded with DiD and FAM-CpG (DiD+FAM-CpG-N/A) showed obvious green and red fluorescence as well as obvious colocalization of the two types of fluorescence (Fig. 2d), implying the successful integration of nanoparticles 1 and CpG. These results were confirmed by agarose gel electrophoresis (Fig. 2e), which indicated the complete encapsulation of CpG into the nanoparticles.

To examine whether the described nanoplatform could immobilize large-molecular-weight proteins, MNPs were incubated with FITC-labeled OVA and analyzed by Western blotting and flow cytometry (Fig. 2f, g). The appearance of both FITC and DiD signals in the DiD+FITC-OVA-N/A group confirmed that MNPs could immobilize simultaneously a nuclei acid and a protein, serving as a universal platform for chemo-immunotherapy-based combination therapy.

Next, we examined whether MNPs could serve as a versatile platform for the immobilization of atezolizumab. Dynamic light scattering measurements showed that the average hydrodynamic diameter of nanoparticles increased by approximately 11 nm after incubation with atezolizumab at 4 °C for 1 h, suggesting the successful immobilization of the antibody (Fig. 2h). The nanoparticle size also increased after incubation, with nanoparticles 3 having the largest size, followed by nanoparticles 2 and 1. In addition, nanoparticles 1 showed a positive surface charge, while nanoparticles 2 and 3 had a negative surface zeta potential due to the anionic CS polymer. The encapsulation efficiency of atezolizumab-coated MNPs co-loaded with PTX, CQ, CpG, and OVA (CpG+OVA+PTX+CQ-N/A) was 98.75 ± 1.6% for PTX and 96.8 ± 2.1% for CQ, while the total drug loading efficiency was around 4.30% for PTX and 0.86% for CQ.

### Cellular uptake, cytotoxicity, and apoptosis of MNP-treated tumor cells

**Fig. 3:**
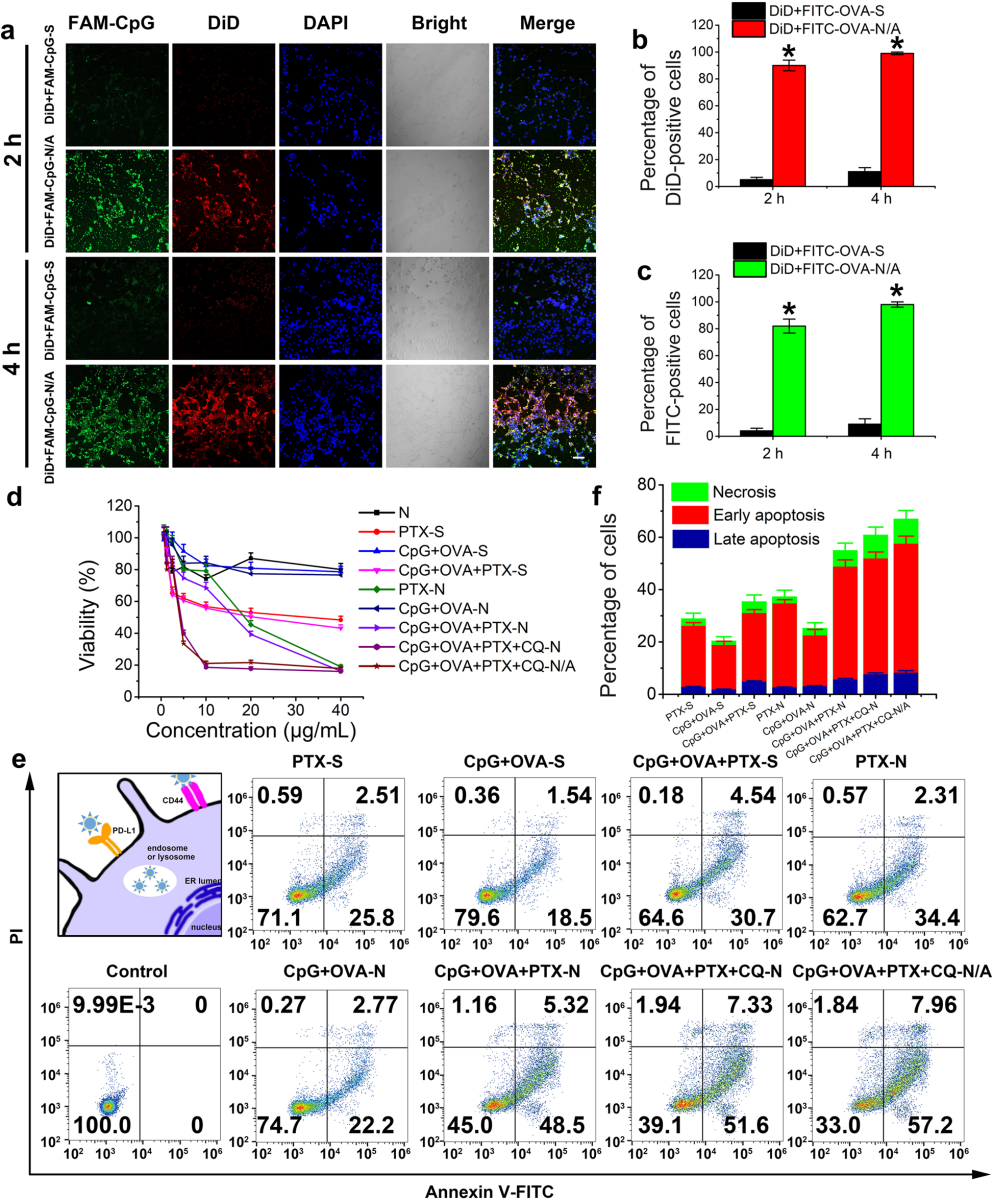
In vitro characterization of multifunctional nanoparticles (MNPs). **a** Confocal microscopy images of 4T1 cells incubated with various formulations for 2 and 4 h. Scale bar, 100 nm. **b**, **c** Number of **b** DiD-positive and **c** FITC-positive 4T1 cells after incubation with DiD+FITC-OVA-N/A and DiD+FITC-OVA-S for 2 and 4 h (DiD, 1 μg/mL). Student’s *t*-test was performed. *P < 0.05. **d** Cell viability of 4T1 cells after incubation with different formulations for 24 h, as determined by the MTT assay. Blank nanoparticles were used as control. **e** Apoptosis of 4T1 cells stained with FITC-Annexin V and propidium iodide (PI) after incubation with different formulations for 24 h. **f** Percentage of cells in the apoptotic or necrotic stage after treatment with different formulations. Data are shown as mean ± SD (*n* = 3). Cells without any treatment were set as control group. CpG, immunopotentiator; CQ, chloroquine; DAPI, 4′,6-diamidino-2-phenylindole; DiD, 4-chlorobenzenesulfonate salt; FAM, fluorescein amidite; FITC, fluorescein; N, nanoparticles, N/A, nanoparticles coated with atezolizumab; OVA, ovalbumin; PTX, paclitaxel; S, solution

To examine the effect of the developed MNPs on the cellular uptake of small and macromolecular drugs, we used the 4T1 cell line that overexpresses both the CD44 and PD-L1 receptors [26]. Confocal microscopy showed that the fluorescence intensity of DiD+FAM-CpG-N/A was stronger than that of a solution containing free DiD and FAM-CpG (DiD+FAM-CpG-S) (Fig. 3a), and that the intensity increased with incubation time from 2 to 4 h. Moreover, the number of DiD-positive cells in the DiD+FITC-OVA-N/A group at 2 h post-incubation was 18 times higher than the number in the DiD+FITC-OVA-S group, and the number increased with incubation time (Fig. 3b). Similar results were observed for FITC-OVA-positive cells in both groups, while the intensity of DiD+FITC-OVA-N/A was found to be nine times higher than that of DiD+FITC-OVA-S (Fig. 3c). These findings indicate that cellular uptake was time-dependent and that MNPs promoted drug penetration, consistent with our previous study [22].

The cytotoxicity of the developed nanoplatform was measured in vitro with MTT assay. Among the tested groups, cells treated with blank nanoparticles showed the highest viability, suggesting that the carrier was non-toxic and caused negligible side effects (Fig. 3d). Moreover, the IC_50_ value was 33.21 μg/mL for free PTX and 22.85 μg/mL for a solution containing free CpG, OVA, and PTX (CpG+OVA+PTX-S). However, after loading onto nanoparticles, the IC_50_ value of PTX-loaded MNPs (PTX-N) and CpG/OVA/PTX-loaded MNPs (CpG+OVA+PTX-N) decreased to 22.18 and 18.12 μg/mL, respectively, reflecting the beneficial effect of the nanoplatform on cytotoxicity. Further loading of CQ reduced the IC_50_ value of CpG+OVA+PTX+CQ-N to 5.54 μg/mL, while atezolizumab immobilization (CpG+OVA+PTX+CQ-N/A) resulted in an IC_50_ value of 5.23 μg/mL (Fig. 3d). These results indicate that the synergistic effect of CQ and PTX along with atezolizumab can significantly enhance the killing effect of individual drugs in cancer cells.

Cells treated with the same formulations as in the cell viability study were then stained with FITC-Annexin V and propidium iodide (PI). Flow cytometry showed that most cells were in early-stage apoptosis or were already dead (Fig. 3e, f). Compared to the PTX-S and CpG+OVA+PTX-S groups, PTX-N and CpG+OVA+PTX-N showed 1.29 and 1.55 times higher ability to induce apoptosis, respectively, implying that MNPs can greatly promote cellular uptake. CpG+OVA+PTX+CQ-N led to higher apoptosis or necrosis rates than CpG+OVA+PTX-N, indicating that CQ can strongly promote cell apoptosis. Furthermore, 67.06% of cells in the CpG+OVA+PTX+CQ-N/A group were in the apoptotic or necrotic stage, which was 1.1 times higher than in the CpG+OVA+PTX+CQ-N group (60.87%) (Fig. 3e, f), suggesting that atezolizumab favors MNP uptake, leading to increased apoptosis.

### Effect of MNPs on autophagosome formation

**Fig. 4:**
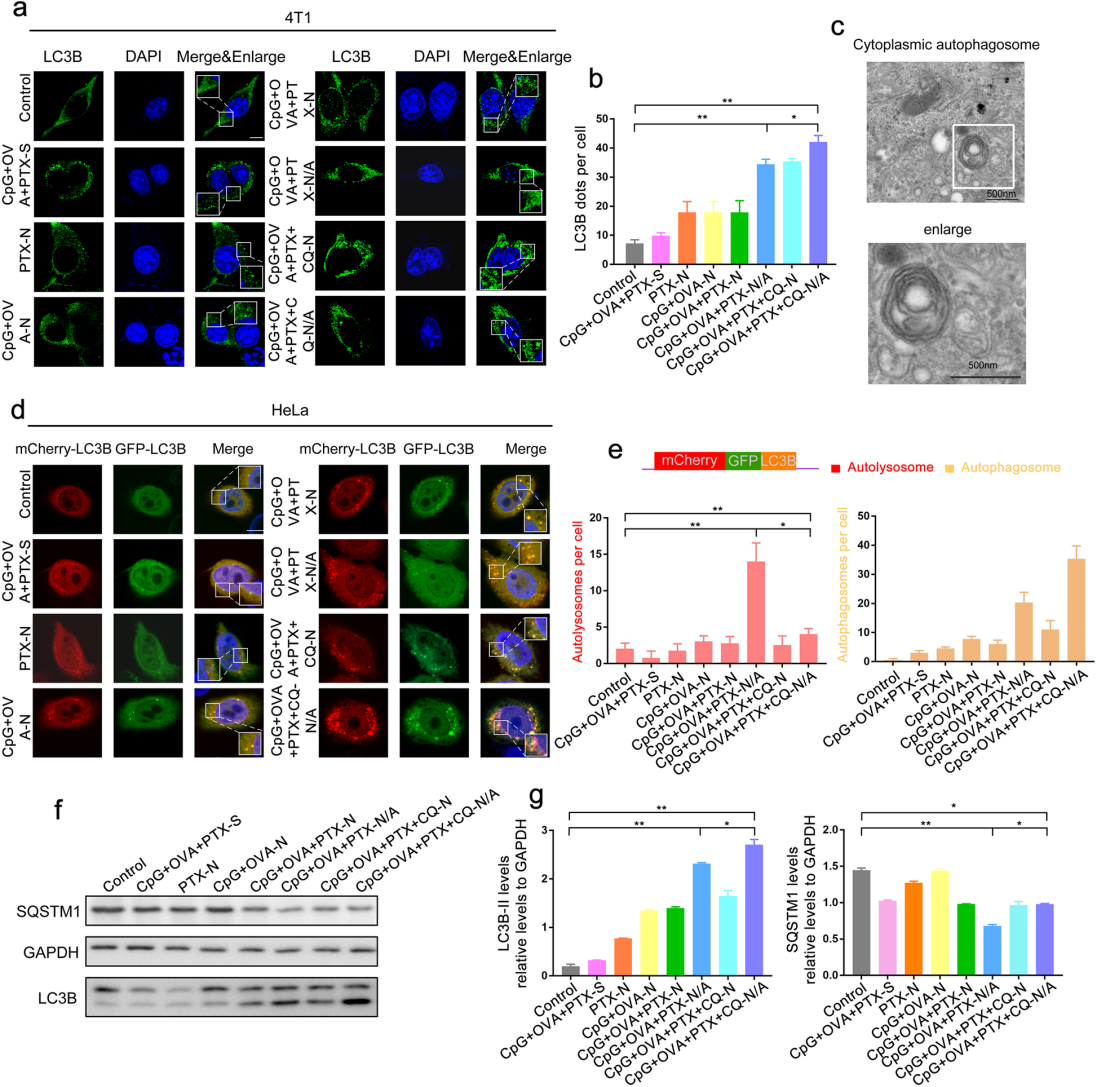
Effect of multifunctional nanoparticles on autophagosome formation. **a** Signals of LC3B puncta detected in 4T1 cells cultured in RPMI-1640 medium with different formulations for 4 h (a 5% glucose solution was used as control), as determined by immunofluorescence assay with an anti-LC3B antibody, followed by confocal microscopy. LC3B-positive puncta were detected in the cytoplasm by fluorescein-conjugated ImmunoPure goat anti-rabbit IgG (green). Cell nuclei were stained with 4′,6-diamidino-2-phenylindole (DAPI). Enlarged boxes highlight LC3B signals. Scale bar: 5 μm. **b** Number of LC3B-positive puncta per cell quantified from ~20 cells treated with different formulations. The means ± SD are from 3 independent experiments. One-way ANOVA was performed. *P < 0.05; **P < 0.01. (*n* = 3 independent experiments.) **c** Transmission electron micrographs of autophagosomes in the cytoplasm of HeLa cells treated with CpG+OVA+PTX-N/A. Scale bar, 500 nm. **d** Confocal microscopy images of HeLa cells transfected with the autophagy dual fluorescent reporter mCherry-GFP-LC3B and cultured with different formulations in Dulbecco’s modified Eagle medium for 4 h. Scale bar: 5 μm. **e** Number of autophagosomes and autolysosomes in HeLa cells treated with different formulations (>15 cells/experiment). Co-localized dots were counted. Data are presented as means ± SD. * *P* < 0.05, ** *P*<0.01 (*n* = 3 independent experiments). **f** Representative Western blots for LC3B-II and SQSTM1. **g** Relative protein levels of LC3B-II and SQSTM1, normalized to levels of GAPDH. Cells without any treatment were set as the control group. Data are shown as mean ± SD (*n* = 3). ^*^P < 0.05, ^**^P < 0.01. CpG, immunopotentiator; CQ, chloroquine; N, nanoparticles; N/A, nanoparticles coated with atezolizumab; OVA, ovalbumin; PTX, paclitaxel; S, solution

The effect of MNPs on autophagosome formation in 4T1 cell lines was evaluated using an immunofluorescence assay with an anti-LC3B antibody, followed by confocal microscopy. Compared to the control group, the number of LC3B-positive puncta increased after treatment with CpG+OVA+PTX-N/A (Fig. 4a, b), indicating that this formulation promoted autophagosome formation. This result was confirmed by transmission electron microscopy of HeLa cells treated with CpG+OVA+PTX-N/A (Fig. 4c). Given that nanomaterial-induced autophagy may promote cancer cell survival [27], cells were then treated with CpG+OVA+PTX+CQ-N/A, which resulted in significant autophagosome puncta accumulation due to the inhibitory effect of CQ (Fig. 4b).

To investigate the mechanism of nanoparticle-associated autophagy, we examined autophagy flux in HeLa cells treated with different formulations using a tandem fluorescent indicator, mCherry-GFP-LC3B (GFP, green fluorescent protein) [28]. Green fluorescence is very sensitive to the acidic environment of lysosomes and quickly quenched in autolysosomes, so red fluorescence was attributed to autolysosomes. CpG+OVA+PTX-N/A significantly promoted autophagosome formation, but even greater autophagosome accumulation was observed in the CpG+OVA+PTX+CQ-N/A group, as CQ inhibited autophagosome–lysosome fusion (Fig. 4d, e).

HeLa cells in DMEM were treated with different formulations to test their ability to induce autophagy. Western blot analysis showed that LC3B-II level was significantly upregulated in the CpG+OVA+PTX-N/A group compared to controls, while its downstream substrate SQSTM1 was downregulated. LC3B-II and SQSTM1 were significantly accumulated in the CpG+OVA+PTX+CQ-N/A group compared to CpG+OVA+PTX-N/A, suggesting that CQ was effective in reducing the flux as evidenced by accumulation of LC3B-II and SQSTM1 (Fig. 4f, g).

### In vivo targeting ability of MNPs

**Fig. 5:**
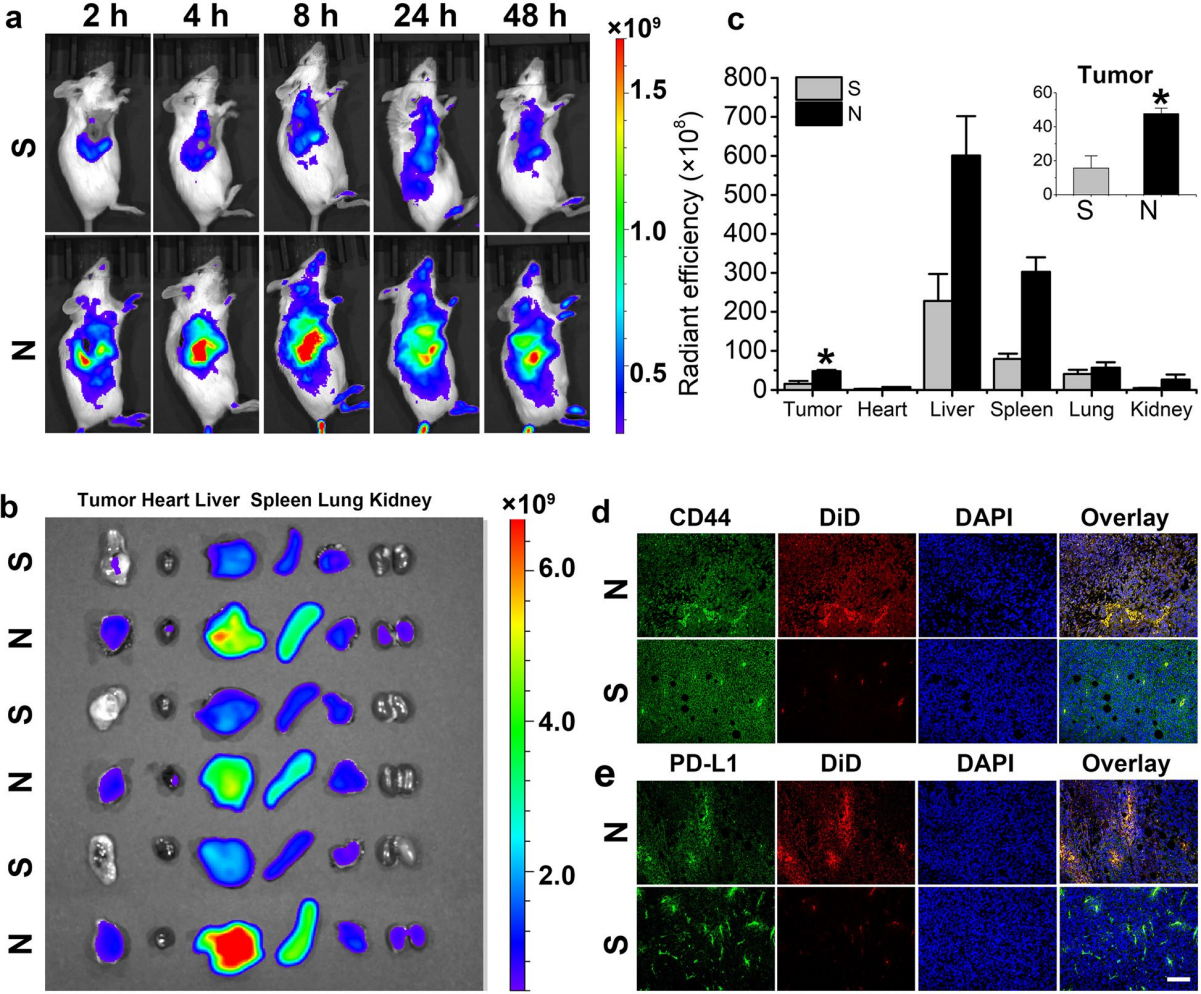
In vivo targeted delivery of multifunctional nanoparticles (MNPs). **a** Fluorescence imaging of 4T1 breast tumor-bearing mice at 2, 4, 8, 24, and 48 h post-administration of DiD-loaded MNPs (N) or a solution of free DiD (S). **b** Nanoparticle distribution in tumors and major organs at 48 h post-injection of DiD-loaded MNPs (N) or a solution of free DiD (S). **c** Semiquantitation of total radiant efficiency in isolated tumors and major organs at 48 h post-injection of DiD-loaded MNPs (N) or a solution of free DiD (S). Data are shown as mean ± SD (*n* = 3). Student’s *t*-test was performed. ^*^P < 0.05. **d**, **e** Confocal imaging of frozen tumor sections. Tumor cells were stained with **d** anti-CD44 antibody (green) or **e** anti-PD-L1 antibody (green). Cell nuclei were stained with 4′,6-diamidino-2-phenylindole (DAPI; blue). Red fluorescence (DiD) indicated MNPs. Mice injected with 5% glucose solution were set as control group. CQ, chloroquine; DiD, 4-chlorobenzenesulfonate salt

The in vivo targeting ability of CpG+OVA+PTX+CQ-N/A was examined by living imaging. At 2 h post-injection, DiD-loaded CpG+OVA+PTX+CQ-N/A (N) showed a stronger fluorescence intensity at the tumor site than a solution of free DiD (S), suggesting that atezolizumab and CS greatly promoted nanoparticle accumulation (Fig. 5a). However, at 24 and 48 h post-injection, the fluorescence intensity decreased in both groups due to body scavenging, although the residual fluorescence intensity of the N group remained higher than that of the S group. Ex vivo imaging of major organs also showed that fluorescence was mainly distributed in the liver, lung, and spleen (Fig. 5b), as the liver and spleen are rich in reticuloendothelial cells, such as macrophages, and express the CD44 receptor, favoring the engulfment of the CD44-responsive MNPs. In addition, the fluorescence intensity at 48 h post-injection was 3.05 times higher in the N group than in the S group, confirming that atezolizumab and CS synergistically promoted the targeting ability of nanoparticles toward 4T1 tumor cells (Fig. 5c). Further staining of tumor slices with CD44 and PD-L1 showed that CpG+OVA+PTX+CQ-N/A emitted stronger fluorescence than the free DiD solution (Fig. 5d), consistent with the in vivo imaging results. These results suggest that the enhanced targeting and penetration ability of the developed MNPs was due mainly to the tumor-homing effect of atezolizumab and CS.

### Anti-tumor effect of MNPs

**Fig. 6:**
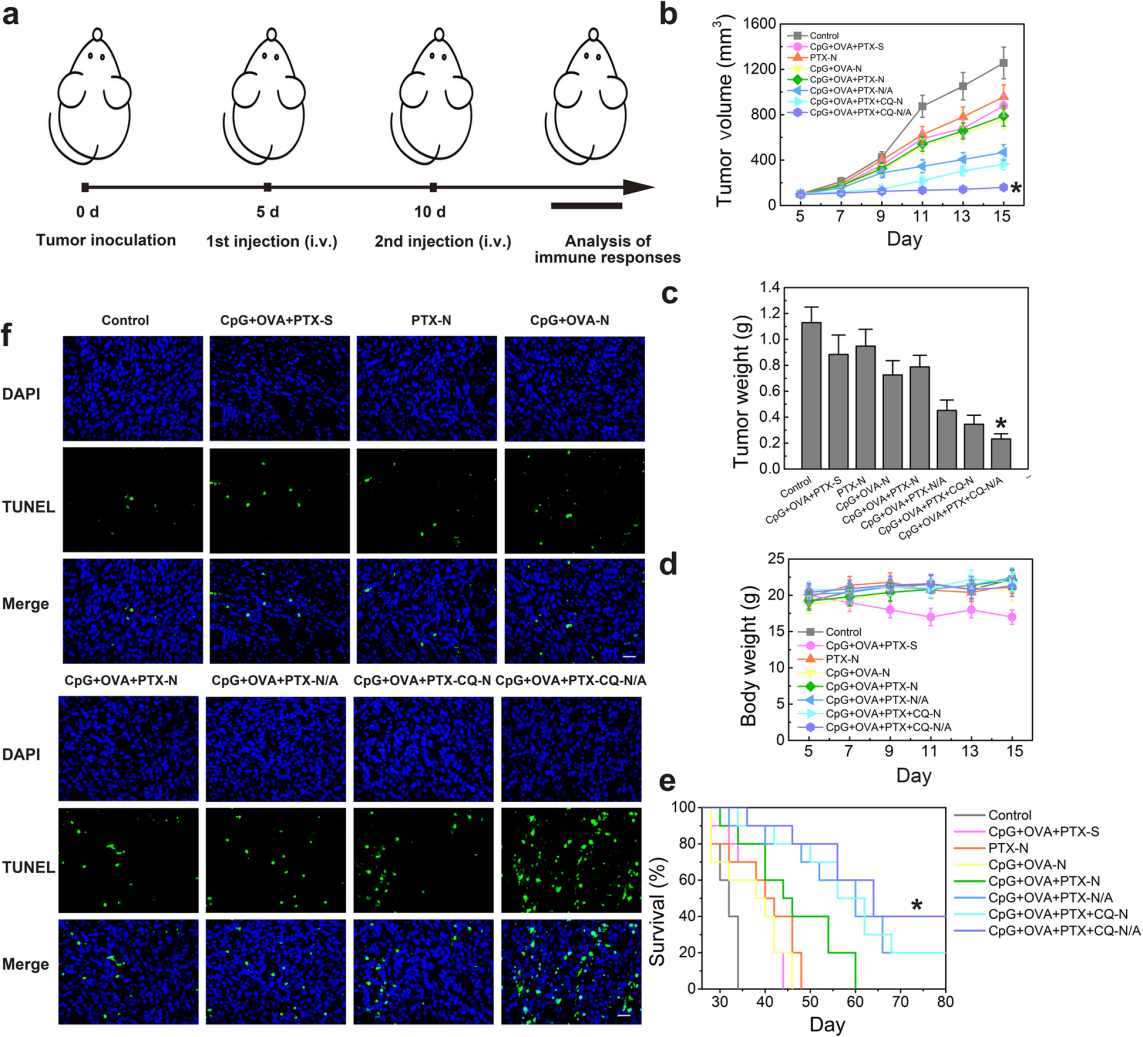
In vivo antitumor efficacy of multifunctional nanoparticles in 4T1 breast tumor-bearing Balb/c mice. **a** Establishment of a subcutaneous 4T1 breast cancer model of Balb/c mice. A 5% glucose solution was used as control. **b** Change in tumor volume over time after treatment with different formulations. Data are shown as mean ± SD (*n* = 5). **c** Weight of excised tumors after the completion of the experiment. Data are shown as mean ± SD (*n* = 5). **d** Body weight of 4T1-bearing mice treated with different formulations for up to 15 days. Data are shown as mean ± SD (*n* = 5). **e** Survival rates of mice over time after treatment with different formulations. Data are shown as mean ± SD (*n* = 10). **f** Tumor sections collected on day 15 after the indicated treatments and visualized by TUNEL labeling. Scale bar, 50 μm. Student’s *t*-test was performed. ^*^P < 0.05 *vs* control. Mice injected with 5% glucose solution were set as control group. CpG, immunopotentiator; CQ, chloroquine; DAPI, 4′,6-diamidino-2-phenylindole; N, nanoparticles; N/A, nanoparticles coated with atezolizumab; OVA, ovalbumin; PTX, paclitaxel; S, solution; TUNEL, terminal deoxynucleotidyl transferase dUTP nick end-labeling

To evaluate the in vivo antitumor effect of the different preparations, we used a subcutaneous 4T1 breast cancer model of Balb/c mice that usually show decreased long-term survival, even after various treatments [5, 29]. On day 0, mice bearing a large breast tumor were subcutaneously injected with 4T1 cells into the right flank. At 5 days post-injection, the tumor volume reached ~50 mm^3^. On days 5 and 10, mice were injected intravenously with different formulations and the antitumor efficacy, immune response, and autophagy response were analyzed (Fig. 6a).

Estimation of the tumor volume in the different treatment groups indicated that CpG+OVA+PTX-S could not suppress tumor growth due to the poor targeting capacity of free drugs (Fig. 6b). In contrast, a stronger suppressive effect was observed in the CpG+OVA+PTX-N group, suggesting that MNPs effectively targeted the tumor site. Although further loading of the nanoplatform with CQ enhanced the tumor-suppressive effect and inhibited autophagy, CpG+OVA+PTX+CQ-N did not completely block tumor growth when compared to CpG+OVA+PTX-N. Conversely, CpG+OVA+CQ+PTX-N/A significantly delayed tumor progression, indicating the beneficial synergistic effect of chemotherapy and immune-checkpoint blockade therapy.

The strongest suppressive effect of CpG+OVA+CQ+PTX-N/A compared to the other nanoformulations was also confirmed in measurements of tumor weight, which was < 0.3 g only for the CpG+OVA+CQ+PTX-N/A group (Fig. 6c). Based on these results, we also calculated the inhibition rates of the different preparations, which revealed several treatments that were ineffective based on poor tumor inhibition rate: PTX-N (16.03%), CpG+OVA-N (35.72%), CpG+OVA+PTX-S (21.71%), and CpG+OVA+PTX-N (30.24%). In contrast, CpG+OVA+PTX+CQ-N reduced tumor volume by 69.45%, which was 2.3 times higher than the reduction achieved by CpG+OVA+PTX-N, indicating that CQ greatly enhanced the therapeutic effect of MNPs. After atezolizumab immobilization (CpG+OVA+PTX+CQ-N/A), the inhibition rate increased further to 79.45%, i.e., 1.15 times higher than CpG+OVA+PTX+CQ-N, highlighting the therapeutic effect of the anti-PD-L1 antibody. This conclusion was confirmed by the tumor inhibition rate of CpG+OVA+PTX-N/A (59.97%), which was 1.98 times higher than that of CpG+OVA+PTX-N (30.24%). Consistent with these results, analysis of the tumor morphology suggested that CpG+OVA+PTX+CQ-N/A had the strongest suppressive effect (Additional file 1: Fig. S1).

The body weight of mice in different groups did not change significantly during treatment (Fig. 6d), suggesting that the developed MNPs were safe and biocompatible. Staining of all major tissues (heart, liver, spleen, lung, kidney) with hematoxylin and eosin (H&E) indicated no obvious toxicity for MNPs in Balb/c mice (Additional file 1: Fig. S2). In addition, hemolysis assay (Additional file 1: Fig. S3), routine blood examination (Additional file 1: Fig. S4), apoptosis (Additional file 1: Fig. S5), and autophagy (Additional file 1: Fig. S6) characterization in the liver and spleen of mice further proved the safety of CpG+OVA+PTX+CQ-N/A for normal organs. Among the treatment groups, CpG+OVA+PTX+CQ-N/A achieved the highest survival rate (Fig. 6e) and caused severe nuclei damage and cytosol degradation in tumor cells, as determined by co-staining with terminal deoxynucleotidyl transferase dUTP nick end-labeling (TUNEL) and with 4′,6-diamidino-2-phenylindole (DAPI) (Fig. 6f). Thus, the administration of an autophagy inhibitor along with immunoadjuvants and an immune-checkpoint inhibitor is a more effective cancer treatment strategy than the individual therapies.

### Effect of autophagy on the anticancer activity of MNPs

**Fig. 7:**
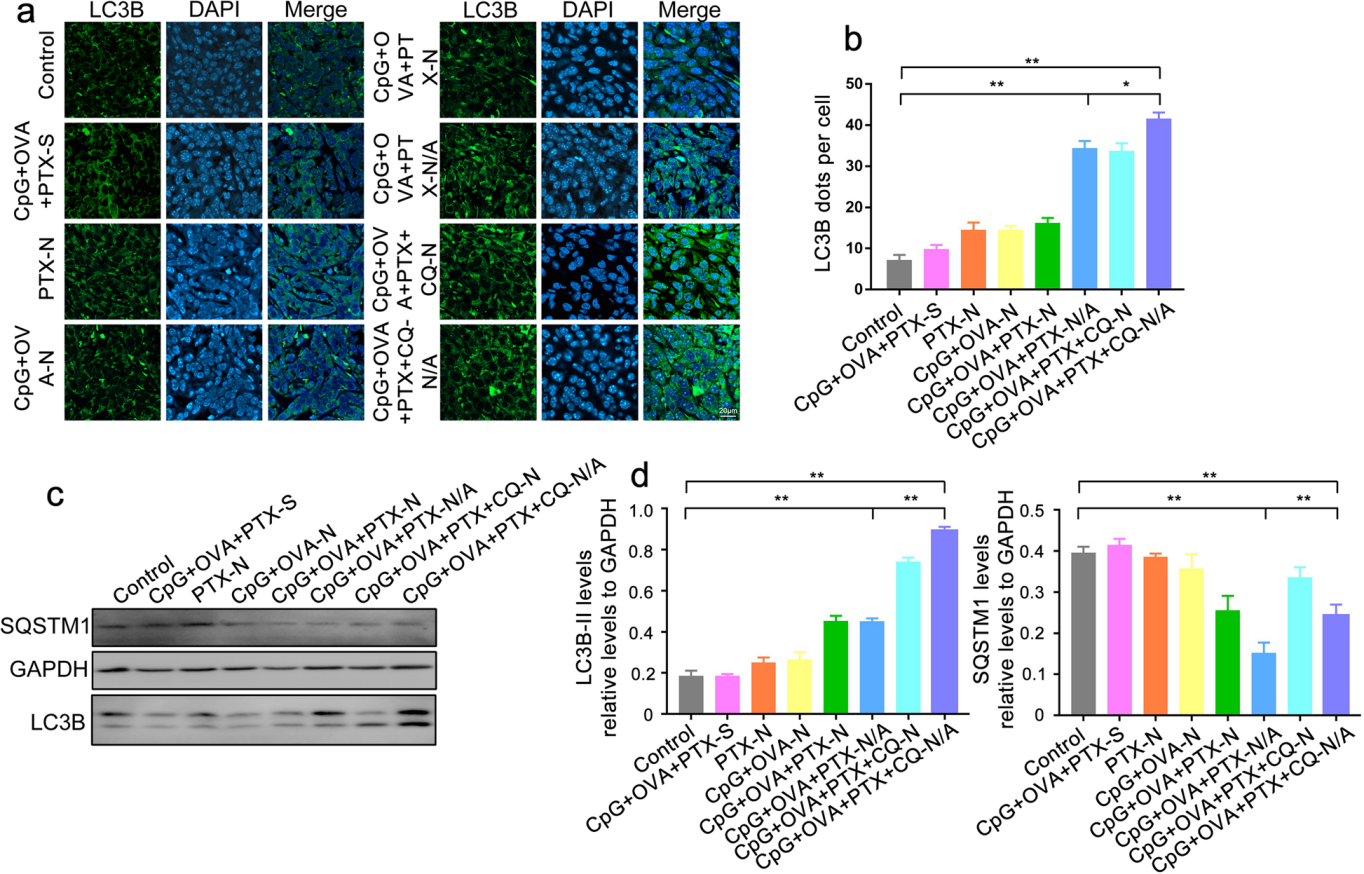
Autophagy inhibition in the tumor enhances the anticancer activity of MNPs. **a** LC3B-positive puncta, as detected by immunofluorescence assay with an anti-LC3B antibody, followed by confocal microscopy. Cell nuclei were stained with 4′,6-diamidino-2-phenylindole (DAPI; blue). Scale bar, 20 μm. **b** Number of LC3B-positive puncta per cell quantified from ~20 cells treated with different formulations. **c** Representative Western blots for SQSTM1 and LC3B-II. **d** Relative protein levels of LC3B-II and SQSTM1, normalized to levels of GAPDH. Data are shown as mean ± SD (*n* = 3). One-way ANOVA was performed. ^*^P < 0.05, ^**^P < 0.01. Mice injected with 5% glucose solution were set as the control group. CpG, immunopotentiator; CQ, chloroquine; N, nanoparticles; N/A, nanoparticles coated with atezolizumab; OVA, ovalbumin; PTX, paclitaxel; S, solution

The effect of autophagy on the anticancer activity of MNPs in vivo was assessed by immunofluorescence analysis. Compared to the control group, CpG+OVA+PTX-N/A significantly promoted autophagosome formation, as indicated by the increased number of LC3B-positive puncta. In addition, autophagy inhibitor CQ was added into MNP created as CpG+OVA+PTX+CQ-N/A which led to significantly higher autophagosome accumulation than CpG+OVA+PTX-N/A, indicating that inhibition of autophagy flux in the tumor greatly favors autophagosome accumulation (Fig. 7a, b). Western blot analysis also confirmed that CpG+OVA+PTX-N/A significantly upregulated LC3B-II compared to the control group, while CpG+OVA+PTX+CQ-N/A promoted the accumulation of LC3B-II at the tumor site (Fig. 7c, d), confirming that autophagy inhibition enhances the anticancer activity of MNPs.

### In vivo immune response

**Fig. 8:**
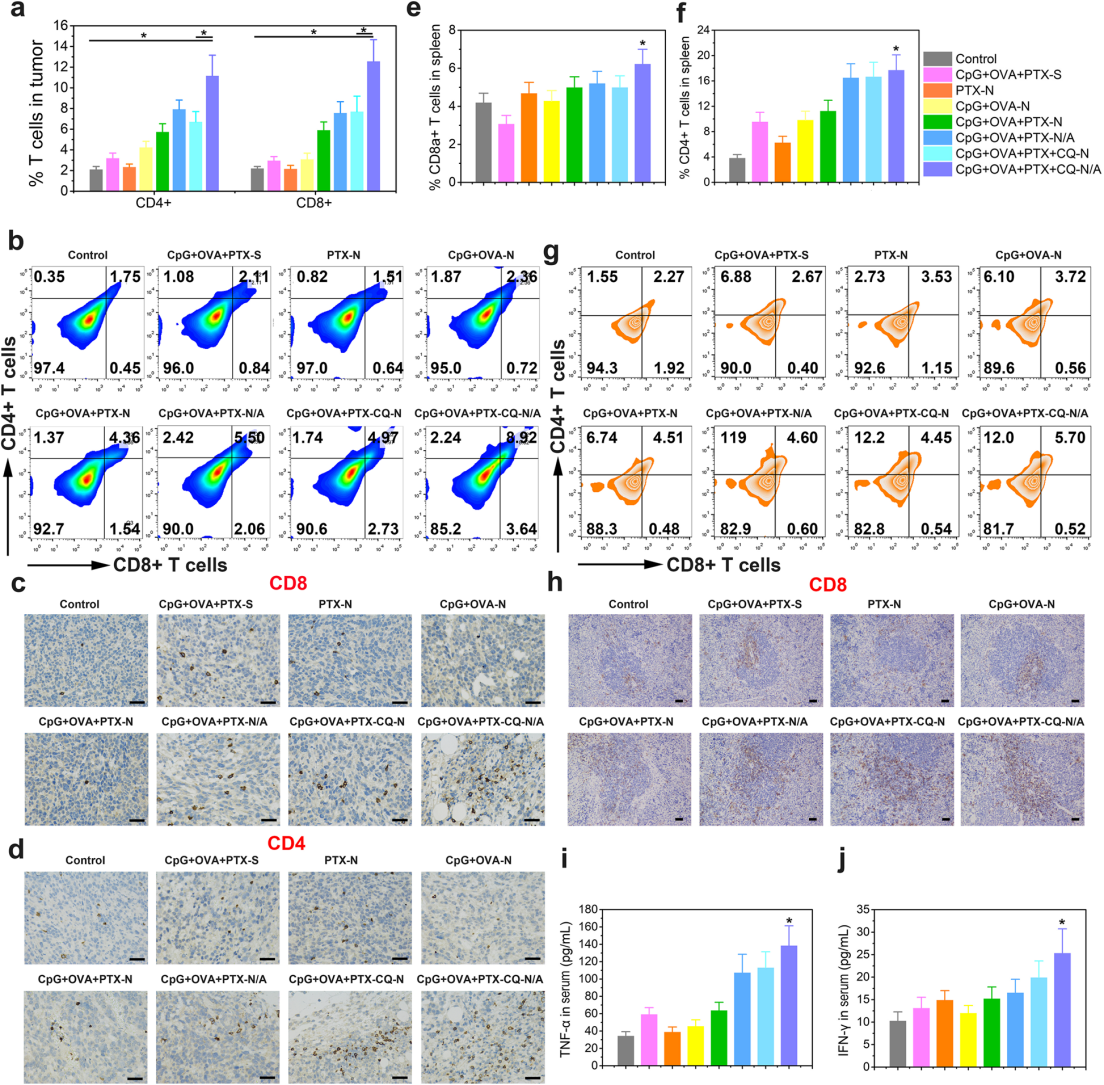
CpG+OVA+PTX+CQ-N/A induce antitumor immune responses *in vivo*. **a** Levels of CD8+ and CD4+ T cells, as determined by flow cytometry on day 15 after the indicated treatments. **b** Representative flow cytometric plots of CD8+ and CD4+ T cells in tumors. **c**, **d** Immunohistochemistry on tumor biopsies. Brown regions indicate the presence of **c** CD8+ T cells and **d** CD4+ T cells. Scale bar, 100 μm. **e**, **f** Levels of **e** CD8+ T cells and **f** CD4+ T cells in the spleen of tumor-bearing mice, as determined by flow cytometry on day 15 after the indicated treatments. **g** Representative flow cytometric plots of CD8+ and CD4+ T cells in the spleen. **h** Immunohistochemical staining of the spleen. Brown regions indicate the presence of CD8+ T cells. Scale bar, 100 μm. **i**, **j** Serum levels of **i** tumor necrosis factor-α (TNF-α) and **j** interferon-γ (IFN-γ) on day 15 after the indicated treatments. Data are shown as ± SD (*n* = 5). One-way ANOVA was performed. ^*^P < 0.05. CpG, immunopotentiator; CQ, chloroquine; N, nanoparticles; N/A, nanoparticles coated with atezolizumab; OVA, ovalbumin; PTX, paclitaxel; S, solution

Cytotoxic T lymphocytes can directly kill cancer cells by releasing perforin, granzymes, and granulysin, while helper T lymphocytes act by regulating adaptive immunity [30]. Here, we determined the levels of CD8+ and CD4+ T cells by flow cytometry and performed immunohistochemical staining to assess the potential immune responses induced by the developed nanoparticles. Compared to the control group, CpG+OVA+PTX+CQ-N/A significantly increased the levels of both cell types (Fig. 8a, b), while immunohistochemistry on tumor biopsies revealed more extensive brown areas in CpG+OVA+PTX+CQ-N/A-treated mice compared to the other treatment groups (Fig. 8c, d, Additional file 1: Fig. S7). Moreover, CpG+OVA+PTX+CQ-N/A increased the number of CD8+ and CD4+ T cells in the spleen by 1.48 and 4.63 times, respectively, compared to the control group (Fig. 8e–g), suggesting that CD8+ and CD4+ T cells are the main effector cells of antitumor response in our animal model. CD3+ and CD8+ T-cell stimulation was confirmed by immunohistochemistry in the spleen (Additional file 1: Fig. S8, Fig. 8h). In contrast, CpG+OVA-N showed reduced immune responses, suggesting that PTX and CQ can lyse tumor cells upon irradiation, serving as tumor-associated antigens.

Our combination therapy not only activated immune cells such as T and natural killer cells, but it also promoted the maturation of dendritic cells and regulated the secretion of cytokines. CpG+OVA+PTX-N, CpG+OVA+PTX+CQ-N, and CpG+OVA+PTX-N/A upregulated the serum levels of tumor necrosis factor-α (TNF-α), which plays an important role in host defense and cellular immunity [31], as well as the levels of interferon-γ (IFN-γ), a mediator of Th1 cells that regulates cell-mediated immune responses [32]. Among these formulations, CpG+OVA+PTX+CQ-N/A nanoparticles led to the greatest increase of TNF-α and IFN-γ (Fig. 8i, j), while they could also considerably reduce the number of PD-L1+ cells compared to control (Additional file 1: Fig. S9), suggesting that they exerted their therapeutic effect by regulating cytokine expression and blocking PD-L1 in T lymphocytes.

Taken together, our experiments suggest that autophagy inhibition in tumors combined with immunoadjuvants and an anti-PD-L1 antibody can enhance immunotherapy of advanced breast cancer, delay tumor growth, and prevent recurrence. Our formulations appear to exert these effects by inhibiting immune evasion of tumor cells *via* immunosuppression reversal and inducing a T cell-mediated antitumor immune response through dendritic cell maturation, upregulation of tumor-associated antigens and cytokines, and generation of T and natural killer cells.

### Immune-memory effect and metastasis inhibition

During T cell proliferation and differentiation, some memory cells are differentiated to exert long-term antitumor effects [33]. To verify the occurrence of long-lasting immune memory in this study, we established a mouse lung metastasis model after removing the primary tumor. On the 10th day of follow-up, severe lung metastasis was observed in the control group, while Ki67 staining confirmed the rapid proliferation of tumor cells in the lungs, which could then trigger systemic spread and lead to death (Additional file 1: Fig. S10a-b). Interestingly, CpG+OVA+PTX+CQ-N/A partially inhibited lung metastasis, reduced lung weight (Additional file 1: Fig. S10c) and the number of lung metastases (Additional file 1: Fig. S10d), and prolonged survival time compared to control. These results strongly suggest that the described MNPs are able to treat primary breast cancer as well as inhibit lung metastasis.

## Discussion

Cancer is a complex and adaptive ecosystem that cannot be easily treated with individual therapies. Although combination therapy based on nanomaterials has recently attracted particular attention [34–36], the effective delivery of nanomedicines with sufficient concentration in malignant cells depends strongly on their transport properties under hypoxia and avascular conditions [37]. Therefore, recent studies on anticancer therapy have focused on the development of biomaterials for targeted drug delivery [38]. In this study, we used a simple, environmentally friendly method and a PEI-OA polymer complex to construct the first PD-L1- and CD44-responsive multifunctional nanoplatform that can be loaded simultaneously with two chemotherapeutic drugs, an antigen, an immunopotentiator, and an immune checkpoint inhibitor. In vitro and in vivo studies of the developed MNPs indicate that this combination treatment strategy can strongly inhibit tumor growth and prevent relapse and lung metastasis of advanced breast cancer.

Recent preclinical studies at the interface of biomaterial science, drug delivery, and cancer vaccines have highlighted the promising potential of combining different types of cancer vaccines (e.g., DNA, mRNA, peptide/protein, or cell-based) with various delivery systems, such as nanoparticles, microparticles, self-assembled materials, and biomaterial scaffolds [39–41]. Biomaterial-based cancer vaccines have also been found to be more effective than conventional vaccines [42, 43], as they can be delivered to the body in a controlled manner, while tuning of their targeting moieties and physical properties, such as size, shape, charge, or porosity, can achieve selective delivery to target cells and tissues with desirable drug release kinetics [44].

In the present work, we selected PEI-OA as the nanoplatform core due to its amphiphilicity, positive charge, and good biocompatibility [22]. PEI derivatives have also been shown to stimulate multiple damage-associated molecular pattern receptors and exert potent adjuvanticity, thus promoting dendritic cell activation and cross-presentation [45]. To avoid off-target toxicity and increase the in vivo safety of the described MNPs, the inner core was further modified with atezolizumab and CS. Using a model of mice bearing large breast tumors, we found that two doses of MNPs loaded with OVA and CpG inhibited 35.72% of the established tumors. These results suggest that our approach may be effective against large tumors, which are generally not easily permeable to drugs or delivery materials [46]. Although immunotherapy may be a promising treatment strategy against breast cancer, most patients respond poorly due to T-cell anergy [47]. Therefore, most recent studies have employed immune-checkpoint inhibitors, such as PD-1/PD-L1 antibodies, combined with other therapeutic treatments, to improve therapeutic effects [48]. Based on this, our MNPs were further loaded with an anti-PD-L1-antibody to block PD-L1-mediated immune responses, offering a long-term therapeutic effect in the immunosuppressive TME, which is a less expensive and potentially safer approach than chimeric antigen receptor T-cell immunotherapy [49].

TNF-α and IFN-γ are cytokines that play an active role in the anti-tumor immune responses. TNF-α, a mediator of cellular immunity, can kill tumor cells directly without harming normal cells [50]. TNF-α is found in the TME that is involved in all stages of breast cancer development, affecting tumor cell proliferation and survival, epithelial-to-mesenchymal transition (EMT), metastasis, and recurrence [51]. IFN-γ is a critical effector molecule to tumor rejection [52]. IFN-γ can impede tumor growth by acting directly on cancer cells; or induced regression of the tumor vasculature, resulting in arrest of blood flow and subsequent collapse of tumors; or drives the fragility of surrounding Tregs, boosts antitumor immunity, and facilitates tumor clearance [53]. The high production of TNF-α and IFN-γ induced by CpG+OVA+PTX+CQ-N/A (Fig. 4e) enlightened us that CpG+OVA+PTX+CQ-N/A could be a valuable vector for antitumor vaccines. We next assessed the CD8+ T cell response induced by CpG+OVA+PTX+CQ-N/A nanoparticles. Tumors and spleens of vaccinated mice were harvested on day 15 and homogenized into a cell suspension; proportions of antigen-specific, CD4+ and CD8+ T cells were determined. CpG+OVA+PTX+CQ-N/A nanoparticles induced a stronger Th1 cell (CD4+) and cytotoxic CD8+ T cell response than other groups in tumors, higher proportion of CD8+ T cells in spleens (Fig. 8g, h). The production of IFN-γ (Fig. 8i) and TNF-α (Fig. 8j) in lymph nodes and spleens also indicated stronger CD8+ T cell responses.

Our results also suggested that, in the early stages of treatment, PTX lysed most of the tumor cells, which then acted as tumor-associated antigens capable of stimulating a strong antigen-specific immune response. PTX mediated enhancement of antitumor immunity results, at least in part, from the stimulation of dendritic cells by dead or dying tumor cells induced by PTX. Recent advances have demonstrated that some chemotherapeutics including PTX could act as immunogenic cell death inducers by arousing calreticulin exposure on tumor cell surface and the release of high mobility group box 1 from tumor cells [54, 55]. In addition, concurrent inhibition of autophagy with CQ prevented tumor relapse for a longer time than free chemotherapeutic drugs did. The described killing effect was achieved with only two injections of CpG+OVA+PTX+CQ-N/A and resulted in the downregulation of PD-L1 in T cells and the induction of CD8+ and CD4+ T cell generation, which are probably the main effector cells for tumor regression and recurrence.

The present study clearly shows that the developed MNPs not only accumulate selectively at the tumor site, but can also penetrate deep into large tumors, where blood vessels are sparse and oxygen levels are low. In addition, our results on immune responses after combination therapy provide important insights into the crosstalk between biomaterial-mediated therapy and cancer immunotherapy.

## Conclusions

We used PEI-OA to construct an autophagy-responsive nanoplatform that efficiently encapsulates two hydrophobic chemotherapeutic drugs for enhanced breast cancer treatment. Co-loading of the anti-PD-L1 antibody provides MNPs with favorable tumor targeting and permeating properties and enhances their ability to inhibit the growth of primary breast cancer, while CS modification promotes their accumulation at the tumor site. CpG+OVA+PTX+CQ-N/A showed high cytotoxicity against 4T1 cells and significantly improved the levels of CD8+ and CD4+ T cells at the tumor site. In addition, the combination therapy showed long-lasting immune memory and effectively inhibited lung metastasis. CpG+OVA+PTX+CQ-N/A show promise for breast cancer immunotherapy and may serve as a guide for the development of novel drug delivery systems for combined active targeting, autophagy inhibition, and chemotherapy, as well as for the co-delivery of biomacromolecules and small-molecule drugs.

## Supplementary Information

**Additional file 1: Figure S1.** Photographs of tumors. **Figure S2.** Hematoxylin and eosin staining of sectioned heart, liver, spleen, lung, and kidney tissues. **Figure S3.** Hemolysis assay. **Figure S4.** Routine blood examination. **Figure S5.** Apoptosis assay in liver and spleen by western blot. **Figure S6.** Autophagy assay in liver and spleen by western blot. **Figure S7.** CD3+ T cells in mouse tumors by immunohistochemical staining. **Figure S8.** CD3+ T cells in mouse spleen by immunohistochemical staining. **Figure S9.** PD-L1 immunohistochemistry. **Figure S10.** Immune-memory effect and metastasis inhibition.

## Data Availability

The data supporting the findings of this study are available from the corresponding author upon reasonable request.

## Abbreviations

A: Atezolizumab
BCA: Bicinchoninic acid
BSA: Bovine serum albumin
CpG: Immunopotentiator
CQ: Chloroquine
CS: Chondroitin sulfate
DAPI: 4′,6-Diamidino-2-phenylindole
DC: Dendritic cell
DiD: 4-Chlorobenzenesulfonate salt
DMEM: Dulbecco’s modified Eagle medium
FAM: Fluorescein amidite
FITC: Fluorescein
H&E: Hematoxylin and eosin
HS15: Solutol HS15®
IFN-γ: Interferon-γ
MHC: Major histocompatibility complex
MNP: Multifunctional nanoparticles
N: Nanoparticles
N/A: Nanoparticles coated with atezolizumab
OA: Oleic acid
OVA: Ovalbumin
PEI: Polyethyleneimine
PEI-OA: Polyethyleneimine-oleic acid polymer
PI: Propidium iodide
PTX: Paclitaxel
S: Solution
SD: Standard deviation
TME: Tumor microenvironment
TNF-α: Tumor necrosis factor-α
TUNEL: Terminal deoxynucleotidyl transferase dUTP nick end-labeling

## References

[1] Kuang J, Song W, Yin J, Zeng X, Han S, Zhao YP (2018). iRGD modified chemo-immunotherapeutic nanoparticles for enhanced immunotherapy against glioblastoma. Adv Func Mater..

[2] Du H, Zhao S, Wang Y, Wang Z, Chen B, Yan Y (2020). pH/Cathepsin B hierarchical-responsive nanoconjugates for enhanced tumor penetration and chemo-immunotherapy. Adv Funct Mater..

[3] Wang H, Liang Y, Yin Y, Zhang J, Su W, White AM (2021). Carbon nano-onion-mediated dual targeting of P-selectin and P-glycoprotein to overcome cancer drug resistance. Nat Commun..

[4] Song C, Phuengkham H, Kim YS, Dinh VV, Lee I, Shin IW (2019). Syringeable immunotherapeutic nanogel reshapes tumor microenvironment and prevents tumor metastasis and recurrence. Nat Commun..

[5] Deng C, Zhang Q, Jia M, Zhao J, Sun X, Gong T (2019). Tumors and their microenvironment dual-targeting chemotherapy with local immune adjuvant therapy for effective antitumor immunity against breast cancer. Adv Sci..

[6] Hu F, Song D, Yan Y, Huang C, Shen C, Lan J (2021). IL-6 regulates autophagy and chemotherapy resistance by promoting BECN1 phosphorylation. Nat Commun..

[7] Feng J, Zhang Y, She X, Sun Y, Fan L, Ren X (2019). Hypermethylated gene ANKDD1A is a candidate tumor suppressor that interacts with FIH1 and decreases HIF1α stability to inhibit cell autophagy in the glioblastoma multiforme hypoxia microenvironment. Oncogene..

[8] Haq S, Wang H, Grondin J, Banskota S, Marshall JK, Khan II (2021). Disruption of autophagy by increased 5-HT alters gut microbiota and enhances susceptibility to experimental colitis and Crohn’s disease. Sci Adv..

[9] Young TM, Reyes C, Pasnikowski E, Castanaro C, Wong C, Decker CE (2020). Autophagy protects tumors from T cell–mediated cytotoxicity via inhibition of TNFα-induced apoptosis. Sci Immunol..

[10] Qiao Y, Choi JE, Tien JC, Simko SA, Rajendiran T, Vo JN (2021). Autophagy inhibition by targeting PIKfyve potentiates response to immune checkpoint blockade in prostate cancer. Nat Cancer..

[11] Xia H, Green DR, Zou W (2021). Autophagy in tumour immunity and therapy. Nat Rev Cancer..

[12] Chen Y, Zhao H, Liang W, Jiang E, Zhou X, Shao Z (2022). Autophagy regulates the cancer stem cell phenotype of head and neck squamous cell carcinoma through the noncanonical FOXO3/SOX2 axis. Oncogene..

[13] Li ZL, Zhang HL, Huang Y, Huang JH, Sun P, Zhou NN (2020). Autophagy deficiency promotes triple-negative breast cancer resistance to T cell-mediated cytotoxicity by blocking tenascin-C degradation. Nat Commun..

[14] Poillet PL, Sharp DW, Yang Y, Laddha SV, Ibrahim M, Bommareddy PK (2020). Autophagy promotes growth of tumors with high mutational burden by inhibiting a T-cell immune response. Nat Cancer..

[15] Xu X, Araki K, Li S, Han JH, Ye L, Tan WG (2014). Autophagy is essential for effector CD8+ T cell survival and memory formation. Nat Immunol..

[16] Amaravadi RK, Kimmelman AC, Debnath J (2019). Targeting autophagy in cancer: recent advances and future directions. Cancer Discov..

[17] Ligeon LA, Pena FM, Vanoaica LD, Núñez NG, Talwar D, Dick TP (2021). Oxidation inhibits autophagy protein deconjugation from phagosomes to sustain MHC class II restricted antigen presentation. Nat Commun..

[18] Zhang Y, Sha R, Zhang L, Zhang W, Jin P, Xu W (2018). Harnessing copper-palladium alloy tetrapod nanoparticle-induced pro-survival autophagy for optimized photothermal therapy of drug-resistant cancer. Nat Commun..

[19] Maycotte P, Aryal S, Cummings CT, Thorburn J, Morgan MJ, Thorburn A (2012). Chloroquine sensitizes breast cancer cells to chemotherapy independent of autophagy. Autophagy..

[20] Paola P, Angela S, Chiara Z (2014). Acidic extracellular pH neutralizes the autophagy-inhibiting activity of chloroquine. Autophagy..

[21] Yamamoto K, Venida A, Yano J, Biancur DE, Kakiuchi M, Gupta S (2020). Autophagy promotes immune evasion of pancreatic cancer by degrading MHC-I. Nature..

[22] Luo J, Zhang Z, Zeng Y, Dong Y, Ma L (2021). Co-encapsulation of collagenase type I and silibinin in chondroitin sulfate coated multilayered nanoparticles for targeted treatment of liver fibrosis. Carbohydr Polym..

[23] Dobrovolskaia MA, Afonin KA (2020). Use of human peripheral blood mononuclear cells to define immunological properties of nucleic acid nanoparticles. Nat Protoc..

[24] Zhang J, Li Y, Liu Q, Huang Y, Li R, Wu T (2021). Sirt6 alleviated liver fibrosis by deacetylating conserved lysine 54 on Smad2 in hepatic stellate cells. Hepatology..

[25] Luo J, Zhang P, Zhao T, Jia M, Yin P, Li W (2019). Golgi apparatus-targeted chondroitin-modified nanomicelles suppress hepatic stellate cell activation for the management of liver fibrosis. ACS Nano..

[26] Chen C, Guo Q, Fu H, Yu J, Wang L, Sun Y (2021). Asynchronous blockade of PD-L1 and CD155 by polymeric nanoparticles inhibits triple-negative breast cancer progression and metastasis. Biomaterials..

[27] Zhang Q, Yang W, Man N, Zheng F, Shen Y, Sun K (2009). Autophagy-mediated chemosensitization in cancer cells by fullerene C60 nanocrystal. Autophagy..

[28] Cheng Y, Lai F, Wang X, Shang D, Zou J, Luo M (2021). Srag regulates autophagy via integrating into a preexisting autophagy pathway in testis. Mol Biol Evol..

[29] Shi X, Yang X, Liu M, Wang R, Qiu N, Liu Y (2021). Chondroitin sulfate-based nanoparticles for enhanced chemo-photodynamic therapy overcoming multidrug resistance and lung metastasis of breast cancer. Carbohydr Polym..

[30] Janjigian YY, Kawazoe A, Yañez P, Li N, Lonardi S, Kolesnik O (2021). The KEYNOTE-811 trial of dual PD-1 and HER2 blockade in HER2-positive gastric cancer. Nature..

[31] Dumitru CD, Ceci JD, Tsatsanis C, Kontoyiannis D, Stamatakis K, Lin JH (2000). TNF-α induction by LPS is regulated posttranscriptionally via a Tpl2/ERK-dependent pathway. Cell..

[32] Fuchs A, Vermi W, Lee J, Lonardi S, Gilfillan S, Newberry R (2013). Intraepithelial type 1 innate lymphoid cells are a unique subset of IL-12- and IL-15-responsive IFN-γ-producing cells. Immunity..

[33] Kaech SM, Tan JT, Wherry EJ, Konieczny BT, Surh CD, Ahmed R (2003). Selective expression of the interleukin 7 receptor identifies effector CD8 T cells that give rise to long-lived memory cells. Nat Immunol..

[34] Uson L, Yus C, Mendoza G, Leroy E, Irusta S, Alejo T (2021). Nanoengineering palladium plasmonic nanosheets inside polymer nanospheres for photothermal therapy and targeted drug delivery. Adv Funct Mater..

[35] Fang H, Guo Z, Chen J, Lin L, Hu Y, Li Y (2021). Combination of epigenetic regulation with gene therapy-mediated immune checkpoint blockade induces anti-tumour effects and immune response in vivo. Nat Commun..

[36] Bosc C, Saland E, Bousard A, Gadaud N, Sabatier M, Cognet G (2021). Mitochondrial inhibitors circumvent adaptive resistance to venetoclax and cytarabine combination therapy in acute myeloid leukemia. Nat Cancer..

[37] Zhang Q, Zhang J, Song J, Liu Y, Ren X, Zhao Y (2021). Protein-based nanomedicine for therapeutic benefits of cancer. ACS Nano..

[38] Lu M, Huang Y (2020). Bioinspired exosome-like therapeutics and delivery nanoplatforms. Biomaterials..

[39] Kalelkar PP, Riddick M, García AJ (2021). Biomaterial-based antimicrobial therapies for the treatment of bacterial infections. Nat Rev Mater..

[40] Balu R, Dutta NK, Dutta AK, Choudhury NR (2021). Resilin-mimetics as a smart biomaterial platform for biomedical applications. Nat Commun..

[41] Wang H, Mooney DJ (2018). Biomaterial-assisted targeted modulation of immune cells in cancer treatment. Nat Mater..

[42] Wang H, Najibi AJ, Sobral MC, Seo BR, Lee JY, Wu D (2020). Biomaterial-based scaffold for in situ chemo-immunotherapy to treat poorly immunogenic tumors. Nat Commun..

[43] Dolgin E (2013). Cancer vaccines: Material breach. Nature..

[44] Roth GA, Picece VCTM, Ou BS, Luo W, Pulendran B, Appel EA (2021). Designing spatial and temporal control of vaccine responses. Nat Rev Mater..

[45] Kievit FM, Veiseh O, Bhattarai N, Fang C, Gunn JW, Lee D (2009). PEI–PEG–chitosan-copolymer-coated iron oxide nanoparticles for safe gene delivery: synthesis, complexation, and transfection. Adv Funct Mater..

[46] Raaijmakers MIG, Rozati S, Goldinger SM, Widmer DS, Dummer R, Levesque MP (2013). Melanoma immunotherapy: historical precedents, recent successes and future prospects. Immunotherapy..

[47] Pulluri B, Kumar A, Shaheen M, Jeter J, Sundararajan S (2017). Tumor microenvironment changes leading to resistance of immune checkpoint inhibitors in metastatic melanoma and strategies to overcome resistance. Pharmacol Res..

[48] Zitvogel L, Kroemer G (2012). Targeting PD-1/PD-L1 interactions for cancer immunotherapy. Oncoimmunology..

[49] Kataoka N, Kunimatsu Y, Tachibana Y, Sugimoto T, Sato I, Tani N (2020). Atezolizumab in combination with carboplatin and etoposide for heavily treated small cell lung cancer. Thorac Cancer..

[50] Cruceriu D, Baldasici O, Balacescu O, Berindan-Neagoe I (2020). The dual role of tumor necrosis factor-alpha (TNF-alpha) in breast cancer: molecular insights and therapeutic approaches. Cell Oncol..

[51] Ma Y, Ren Y, Dai ZJ, Wu CJ, Ji YH, Xu J (2017). IL-6, IL-8 and TNF-α levels correlate with disease stage in breast cancer patients. Adv Clin Exp Med..

[52] Gao Y, Yang J, Cai Y, Fu S, Zhang N, Fu X, Li L (2018). IFN-gamma-mediated inhibition of lung cancer correlates with PD-L1 expression and is regulated by PI3K-AKT signaling. Int J Cancer..

[53] Li Q, Zhou Y, He W, Ren X, Zhang M, Jiang Y, Zhou Z, Luan Y (2021). Platelet-armored nanoplatform to harmonize janus-faced IFN-gamma against tumor recurrence and metastasis. J Control Release..

[54] Duan X, Chan C, Lin W (2019). Nanoparticle-mediated immunogenic cell death enables and potentiates cancer immunotherapy. Angew Chem Int Ed Engl.

[55] Fucikova J, Kepp O (2020). Detection of immunogenic cell death and its relevance for cancer therapy. Cell Death Dis..

