# Supplementary material for: Combination of an autophagy inhibitor with immunoadjuvants and an anti-PD-L1 antibody in multifunctional nanoparticles for enhanced breast cancer immunotherapy
Source: BMC Med. 2022 Oct 28;20:411. doi: 10.1186/s12916-022-02614-8 (PMC9615197; doi:10.1186/s12916-022-02614-8)
Supplement: Supplementary file 1 — Additional file 1: Figure S1. Photographs of tumors. Figure S2. Hematoxylin and eosin staining of sectioned heart, liver, spleen, lung, and kidney tissues. Figure S3. Hemolysis assay. Figure S4. Routine blood examination. Figure S5. Apoptosis assay in liver and spleen by western blot. Figure S6. Autophagy assay in liver and spleen by western blot. Figure S7. CD3+ T cells in mouse tumors by immunohistochemical staining. Figure S8. CD3+ T cells in mouse spleen by immunohistochemical staining. Figure S9. PD-L1 immunohistochemistry. Figure S10. Immune-memory effect and metastasis inhibition. [file 12916_2022_2614_MOESM1_ESM.zip › Additional file 1R3.docx]

Supporting Information

**Combination of an autophagy inhibitor with immunoadjuvants and an anti-PD-L1 antibody in multifunctional nanoparticles for enhanced breast cancer immunotherapy**

Yibin Cheng^1†^, Caixia Wang^2†^, Huihui Wang^1^, Zhiwei Zhang^1^, Xiaopeng Yang^1^, Yanming Dong^1^, Lixin Ma^1*^, and Jingwen Luo^1*^

**
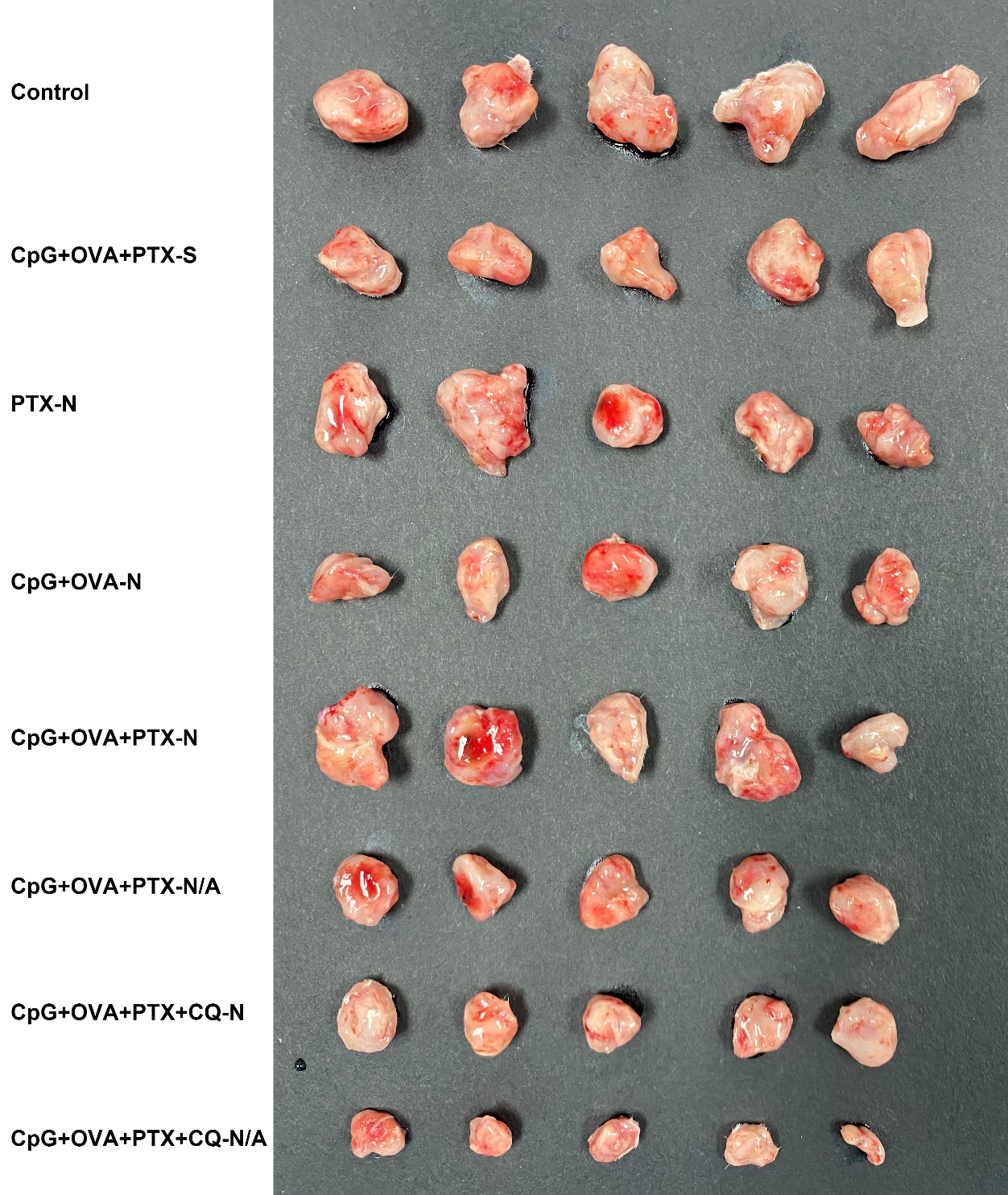
**

**Figure S1.** Photographs of tumors collected on day 15 after treatment with different formulations. 5% glucose solution was used as control. CpG, immunopotentiator; CQ, chloroquine; N, nanoparticles; N/A, nanoparticles coated with atezolizumab; OVA, ovalbumin; PTX, paclitaxel; S, solution.


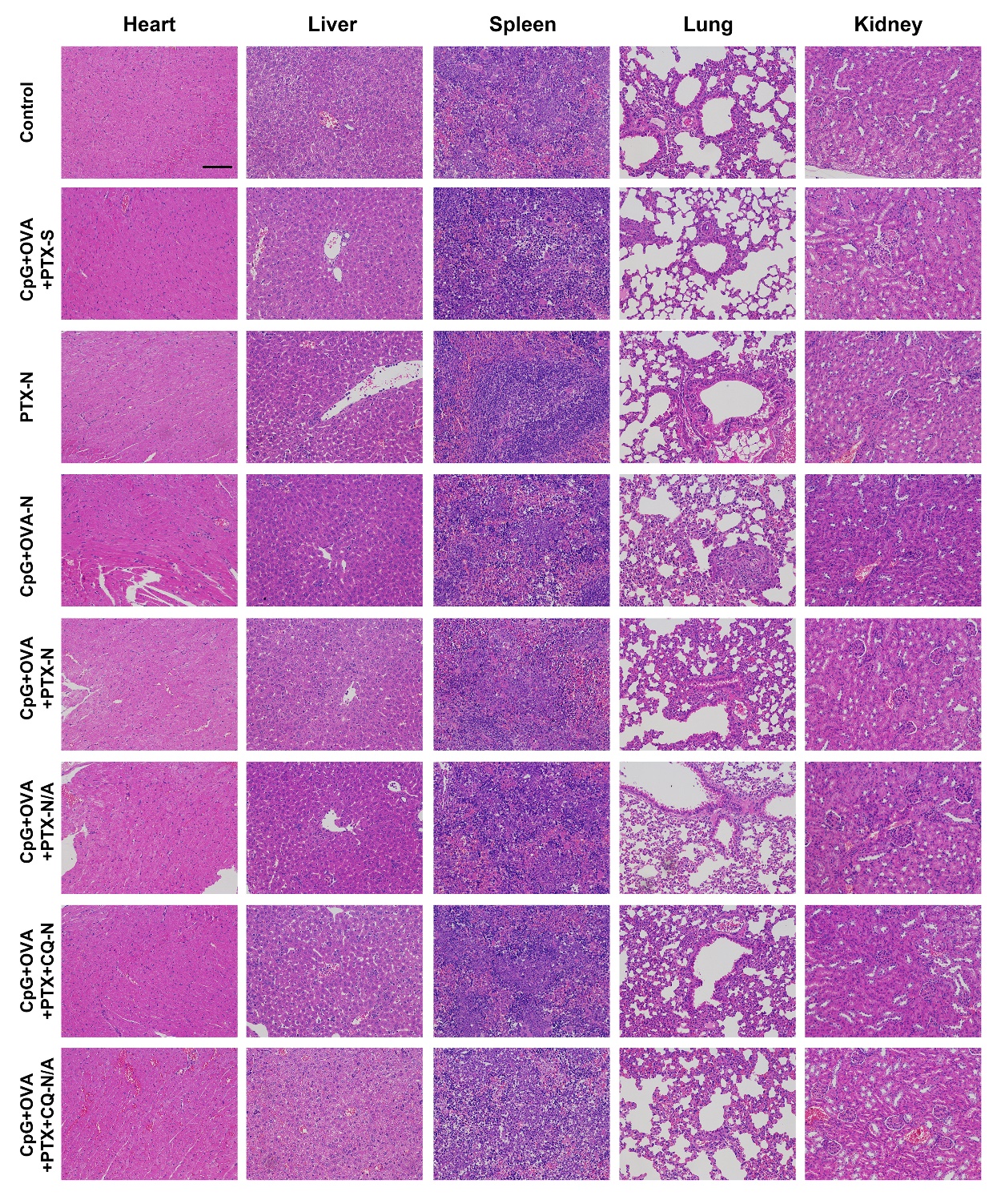


**Figure S2.** Hematoxylin and eosin staining of sectioned heart, liver, spleen, lung, and kidney tissues collected from mice on day 15 after the indicated treatments. 5% glucose solution was used as control. Scale bar, 100 μm. CpG, immunopotentiator; CQ, chloroquine; N, nanoparticles; N/A, nanoparticles coated with atezolizumab; OVA, ovalbumin; PTX, paclitaxel; S, solution.


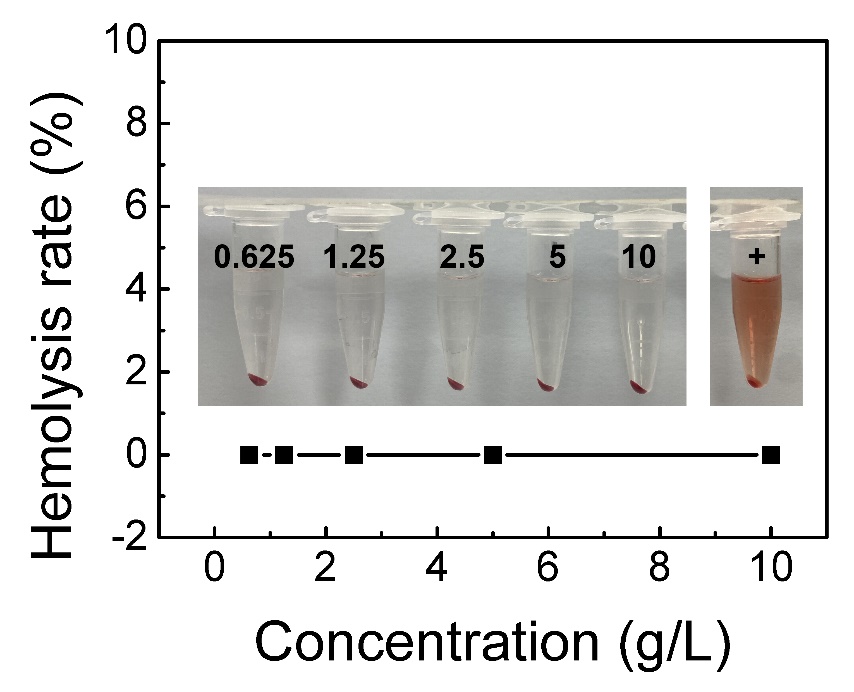


**Figure S3.** Hemolysis assay of CpG+OVA+PTX+CQ-N/A nanoparticles.


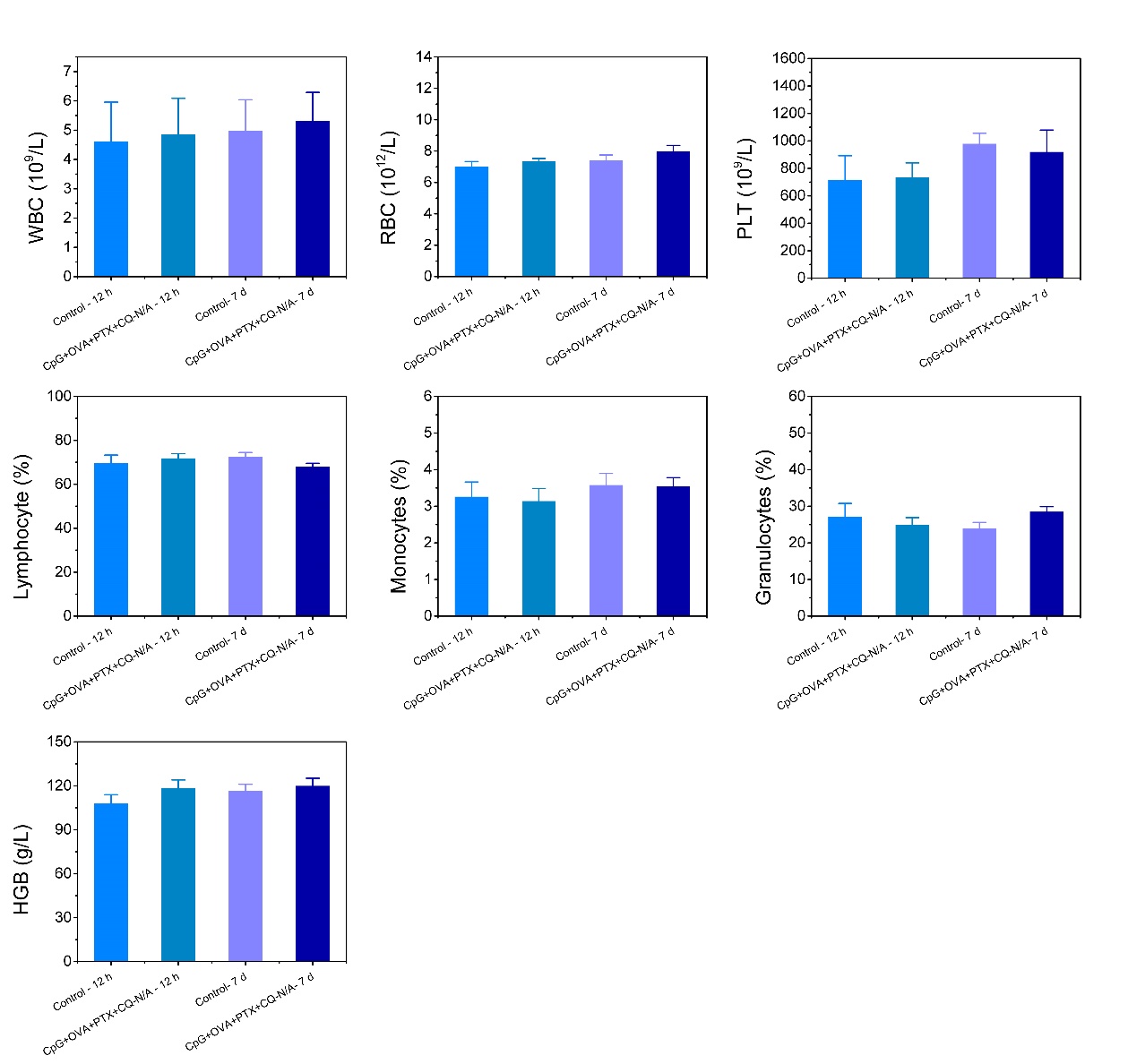


**Figure S4.** Routine blood examination. Mice white blood cells, lymphocytes, monocytes, granulocytes, red blood cells, hemoglobin and platelet levels were determined on day 0.5 and 7 after two CpG+OVA+PTX+CQ-N/A nanoparticles injections.


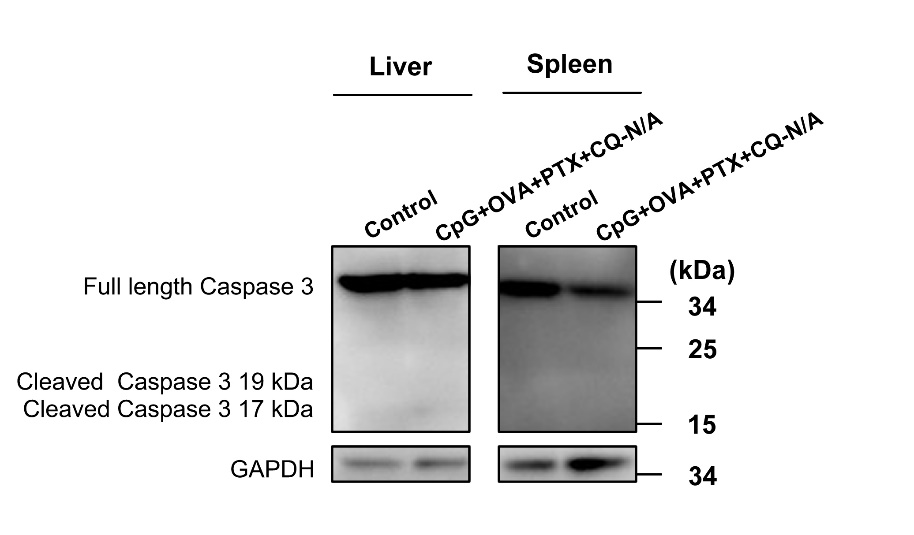


**Figure S5.** Apoptosis assay in liver and spleen by western blot after the indicated treatments.


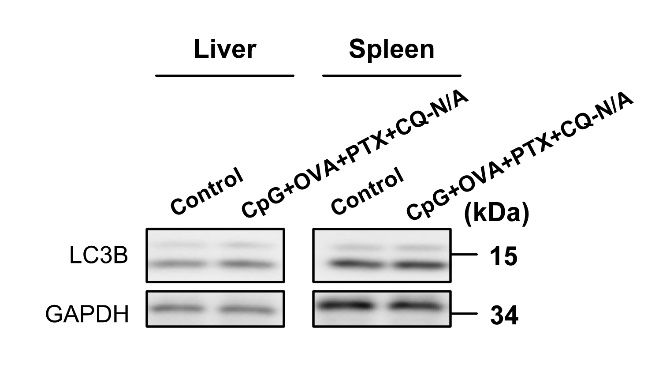


**Figure S6.** Autophagy assay in liver and spleen by western blot after the indicated treatments.


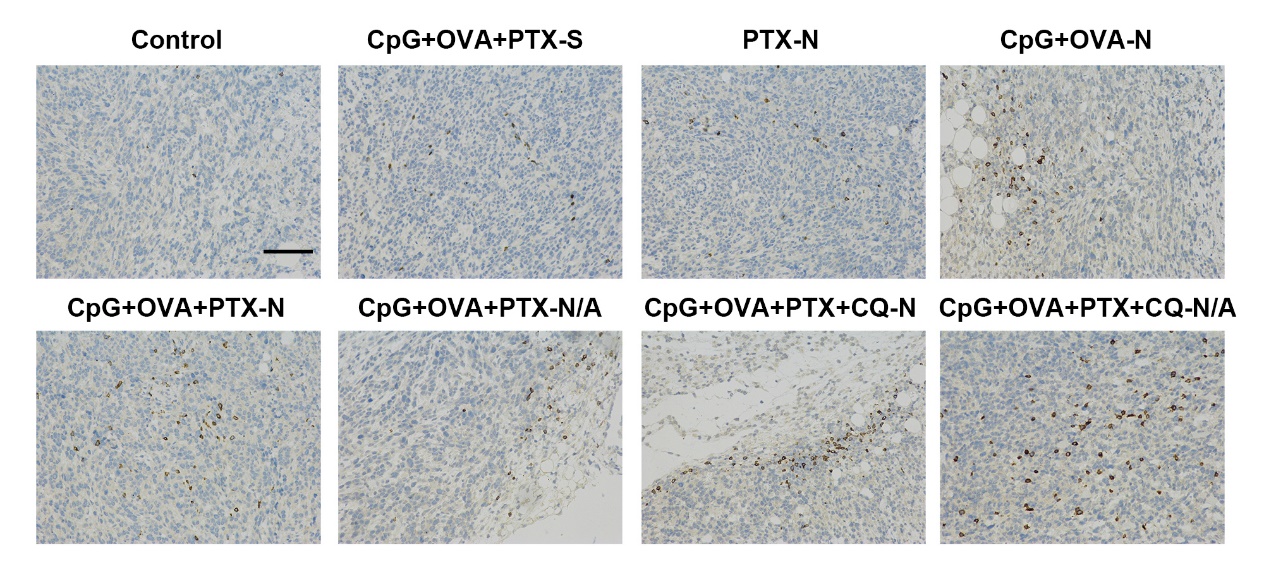


**Figure S7.** CD3+ T cells in mouse tumors by immunohistochemical staining after the indicated treatments. Brown regions indicate the presence of CD3+ T cells. Scale bar, 200 μm.


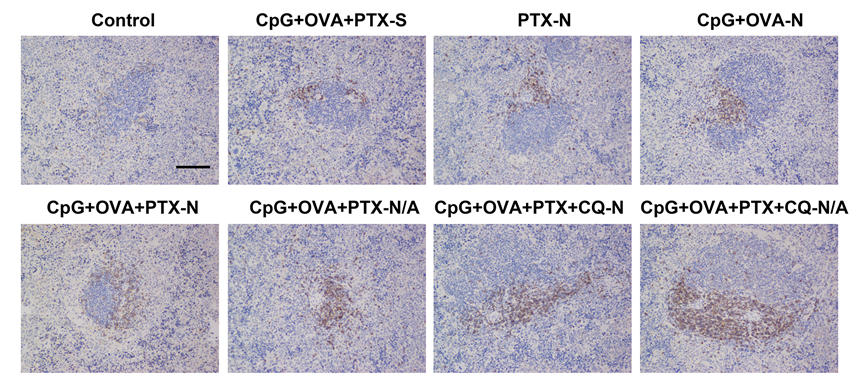


**Figure S8.** CD3+ T cells in mouse spleen by immunohistochemical staining after the indicated treatments. Brown regions indicate the presence of CD3+ T cells. Scale bar, 200 μm.


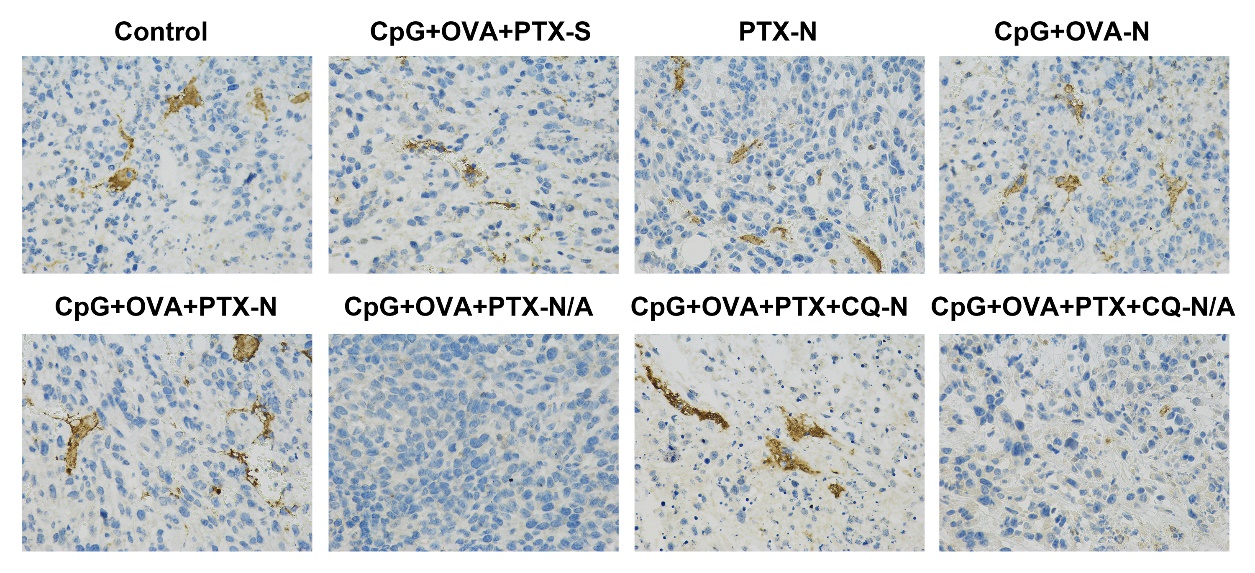


**Figure S9.** PD-L1 immunohistochemistry on day 15 after treatment with different formulations. 5% glucose solution was used as control. CpG, immunopotentiator; CQ, chloroquine; N, nanoparticles; N/A, nanoparticles coated with atezolizumab; OVA, ovalbumin; PTX, paclitaxel; S, solution.


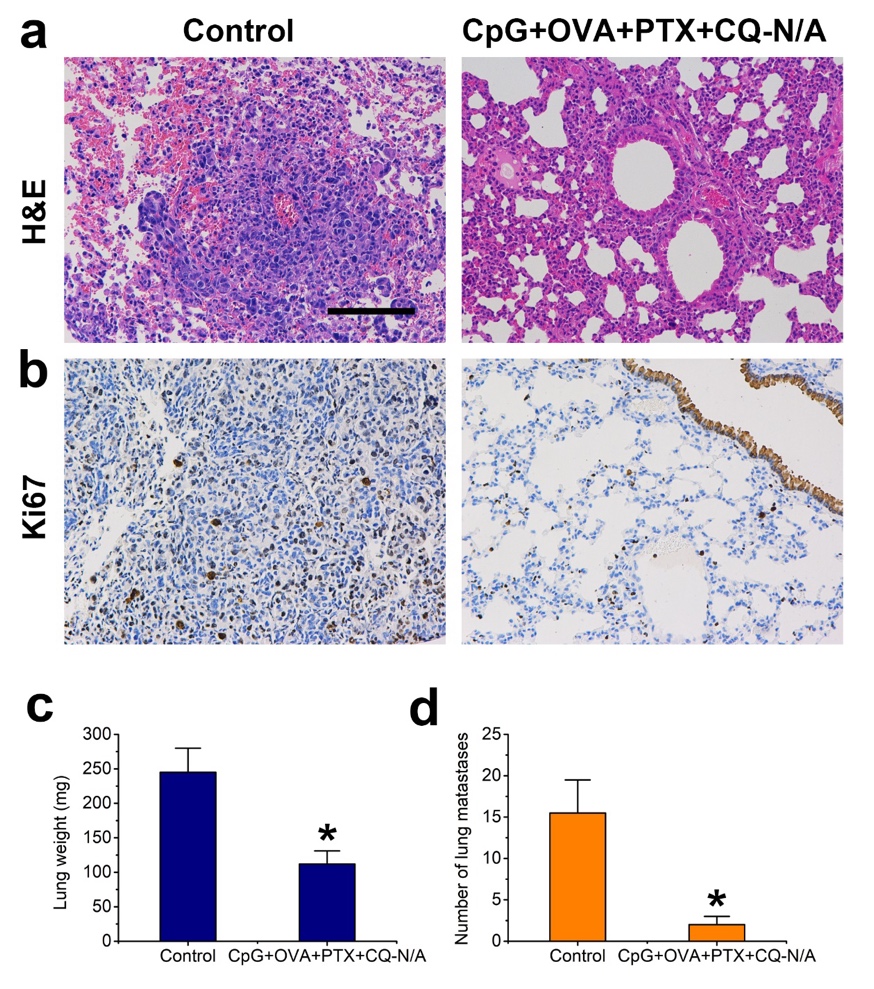


**Figure S10.** (**a**) Staining of lung tissues with hematoxylin-eosin (H&E) after treatment with control and CpG+OVA+PTX+CQ-N/A nanoparticles. (**b**) Ki67 immunohistochemical staining of tumors at 10 days post-treatment with control and CpG+OVA+PTX+CQ-N/A nanoparticles. (**c**) Weight of excised lungs in the control and CpG+OVA+PTX+CQ-N/A groups at the end of treatment. (**d**) Number of lung metastases. Data are shown as mean ± SD (n = 3). ^*^*P* < 0.05. CpG+OVA+PTX+CQ-N/A, atezolizumab-coated multifunctional nanoparticles co-loaded with an immunopotentiator, ovalbumin, paclitaxel, and chloroquine.
