# Supplementary material for: Combination of an autophagy inhibitor with immunoadjuvants and an anti-PD-L1 antibody in multifunctional nanoparticles for enhanced breast cancer immunotherapy
Source: BMC Med. 2022 Oct 28;20:411. doi: 10.1186/s12916-022-02614-8 (PMC9615197; doi:10.1186/s12916-022-02614-8)
Supplement: Supplementary file 1 — Additional file 1: Figure S1. Photographs of tumors. Figure S2. Hematoxylin and eosin staining of sectioned heart, liver, spleen, lung, and kidney tissues. Figure S3. Hemolysis assay. Figure S4. Routine blood examination. Figure S5. Apoptosis assay in liver and spleen by western blot. Figure S6. Autophagy assay in liver and spleen by western blot. Figure S7. CD3+ T cells in mouse tumors by immunohistochemical staining. Figure S8. CD3+ T cells in mouse spleen by immunohistochemical staining. Figure S9. PD-L1 immunohistochemistry. Figure S10. Immune-memory effect and metastasis inhibition. [file 12916_2022_2614_MOESM1_ESM.zip › Additional file 2R3.docx]

Figure 2e:

CPG

Figure 2f:

FITC-OVA

Response to reviewer #3, question 4:

caspase3

 caspase3

GAPDH

 GAPDH

Additional file 1: Fig. S5:

GAPDH+caspase3

Caspase3 long exposure

Additional file 1: Fig. S6:

LC3

GAPDH

Figure 4f:

LC3

P62

GAPDH

Figure 7c:

GAPDH

 P62

LC3
